# Nine Coupled Irrigation–Agronomic Treatments for Water-Saving Rice Production on Albic Soil: An Interpretable Machine-Learning Diagnosis

**DOI:** 10.3390/plants15132037

**Published:** 2026-07-01

**Authors:** Jing Wang, Haomin Wang, Hui Guo, Zhenjiang Si, Tao Liu

**Affiliations:** 1 School of Hydraulic and Electric Power, Heilongjiang University, Harbin 150080, China; 2242161@s.hlju.edu.cn (J.W.); 2232895@s.hlju.edu.cn (T.L.); 2 Heilongjiang Provincial Water Conservancy and Hydropower Survey, Design and Research Institute, Harbin 150080, China; a100e@139.com; 3 MOE Key Laboratory of Groundwater Circulation and Environmental Evolution, China University of Geosciences (Beijing), Beijing 100083, China; 4 School of Water Resources and Environment, China University of Geosciences (Beijing), Beijing 100083, China

**Keywords:** controlled irrigation, soil amendment, water use efficiency, interpretable machine learning, conformal prediction, rhizosphere redox potential, cold-region japonica rice, albic soil

## Abstract

Sustaining rice productivity under the dual constraints of freshwater scarcity and low-temperature stress represents a pressing challenge for high-latitude japonica rice systems worldwide. There is an urgent need to develop coupled irrigation–agronomic management strategies that jointly safeguard yield stability and water use efficiency (WUE) in cold-region rice production. In this study, a two-year field experiment was conducted in 2024–2025 on albic soil (Albic Luvisols, WRB; θfc 38.2% *v*/*v*, pH 5.80, clayey texture with poor permeability and a propensity for subsurface waterlogging) in the Sanjiang Plain, Heilongjiang Province, China (47°15′ N, 133°28′ E), with nine coupled “irrigation regime × auxiliary practice” treatments, comprising conventional continuous flooding, four-level controlled irrigation (CI) at lower thresholds of 60%, 70%, 75%, and 80% θfc, and their combinations with film mulching (FM) or a humic-acid-based soil amendment (SA). An interpretable machine-learning diagnostic framework was developed, with elastic net (EN) as the primary analytical model and random forest (RF) as a nonlinear control, to simultaneously identify core yield predictors and outlier treatments. The principal findings were: (i) The soil-amendment-coupled 75% θfc CI treatment (SACI) increased grain yield by 12.3% and reduced water input by 17.0% relative to conventional continuous flooding, with WUE reaching 1.801 kg m^−3^, a 35.3% gain over the control (*p* < 0.05); these improvements were consistent across both individual years (year × treatment interaction: *p* = 0.601; inter-year rank correlation ρ = 0.967). Lowering the CI threshold below 75% θfc significantly reduced grain yield through diminished effective-panicle retention. (ii) Multi-method consensus analysis (Kendall’s W = 0.871, *p* < 0.01) identified root volume at the milk stage as the most strongly and consistently associated statistical predictor of yield formation, with convergent mechanistic support from independent rhizosphere evidence (Eh, TTC reductive activity). Definitive causal validation awaits isotope-tracing experiments. (iii) The film-mulching × continuous-flooding treatment (FMCG) was diagnosed as a yield-response outlier (permutation test *p* = 0.003), three in situ rhizosphere measurements (redox potential, root TTC-reducing activity, and rhizosphere temperature) supported the proposed mechanism of hot–anoxic rhizospheric inhibition. Methodologically, this study develops a four-level evidence convergence framework that integrates intra-model self-consistency, cross-model (EN vs. RF) consensus, independent rhizosphere evidence, and distribution-free permutation testing, with Jackknife+ conformal prediction and companion Monte Carlo simulations (1000 replicates) used to quantify the reliability boundaries under small-sample conditions (n = 27). These findings provide an evidence-based irrigation–soil co-management strategy for cold-region rice production in Northeast China, and the proposed diagnostic paradigm offers a generalizable, reliability-quantified methodological template for interpretable small-sample modeling in multifactorial coupled field experiments.

## 1. Introduction

Rice feeds more than half of the world’s population, yet consumes 34–43% of global irrigated water [1], with Li et al. [2] estimating the figure at 40%. Remodeling small irrigation and drainage systems in China alone could cut the national rice water footprint by 30%. Rice thus stands at the center of the water–food–ecology nexus central to the UN Sustainable Development Goals. Focusing on China, the northeastern region, as a nationally important food production base, has a core production area, the Sanjiang Plain, which contributes 19% of the world’s total rice production with only 17% of the planted area. However, the region is mainly centered on intensive agricultural production, which has led to the loss of SOD in the black soil zone, intensive heavy industrial activities, concurrent climate change and competition for water between sectors, and increasing pressure on water resources. Liang et al. [3] combined crop distribution data with observed changes in groundwater levels to conclude that the northward expansion of the Sanjiang Plain by 2.19 × 10^6^ ha between 2000 and 2020 coincided with an average decline in the groundwater table of 4.79 m. This is the most striking piece of evidence to date that attributes the expansion of rice to groundwater depletion. The pressure on the region’s resources goes beyond water, as the region is located in a high-latitude cold zone with an annual effective cumulative temperature of only about 2300 °C-d, and the combination of low-temperature stress and freshwater constraints already limits the grouting window. It is against this background that Guo et al. [4] observed in a two-year temperature-controlled field experiment that low-temperature stress at the spike stage caused the most drastic decrease in the net photosynthetic rate of Japonica rice, and consequently suppressed pollen viability and glume fertility, which became a key physiological bottleneck for yield stabilization of Japonica rice in cold regions. Therefore, when freshwater scarcity and cold stress are superimposed on each other, coupled management of irrigation and cultivation to “maintain yield and increase WUE” has become an indispensable agronomic proposition for global sustainable rice cultivation.

For agricultural water conservation, the value of CI and alternating wet and dry irrigation (AWD) has been repeatedly validated by several meta-analyses worldwide. Gao et al. [5] summarized 437 studies covering 93% of the global rice area and showed that CI/AWD reduced irrigation water use by 33.88% and increased WUE, irrigation water use efficiency (IWUE) and water productivity (WP) by 20.27%, 47.58%, and 29.63%, respectively. The mechanism lies in the elevation of inter-root dissolved oxygen and redox potential (Eh), which in turn drives enhanced root oxidative activity and deeper root distribution [6]. However, the yield and water-saving effects of CI/AWD at specific thresholds are clearly stand-dependent and difficult to simply transfer between different soil types. Albic soil in the Sanjiang Plain is characterized by a strongly differentiated profile, in which a bleached, coarse-textured albic horizon overlies a dense, clay-illuvial argic horizon. The low-permeability subsoil simultaneously impedes percolation, causing perched-water subsurface waterlogging, and permits rapid dry-down of the light-textured topsoil. This dual hydraulic behavior contrasts sharply with the more uniform, permeable alluvial and loamy soils, as well as the deep, well-structured black soils, on which most existing CI/AWD evidence has been obtained, so that an identical θfc threshold drives markedly different root-zone water dynamics. However, existing CI/AWD evidence is derived almost entirely from single-factor irrigation regimes in tropical and subtropical systems; multi-year field evidence on albic soil—resolving the CI × auxiliary-practice coupling across a 60–80% θfc gradient and defining the safe lower CI threshold under high-latitude cold conditions—remains lacking.

In terms of supporting cultivation measures, FM and SA are two important types of tools to regulate yield formation in low-temperature rice areas. Mulching can significantly promote yield formation in low-temperature rice areas by elevating root zone temperature. For FM, the yield-enhancing effect stems mainly from warming of the root zone. A meta-analysis by Huang et al. [7], based on a large number of observations of 13 crops in China, showed that on average, FM increased crop yield, WUE, and nitrogen fertilizer bias productivity by about 26%, 33%, and 26%, respectively, and that the increase in yield was significantly higher in cool, semi-arid, and arid zones than in warm and humid zones. The mechanism of yield enhancement by SA is different, as it works mainly by improving soil nutrient supply and utilization. A global meta-analysis by Ma et al. [8] showed that humic acid application increased crop yield, nitrogen use efficiency and nitrogen uptake by 12%, 27% and 17%, respectively, mainly due to the combined effect of elevated cation exchange capacity (CEC) and functional stimulation of microorganisms involved in nitrogen metabolism. It is worth noting that the yield-enhancing effects of FM and SA do not exist in isolation, but often depend on the coordination of irrigation systems. Zhang et al. [6] demonstrated that the coupling of biochar and CI can synchronize the improvement of root morphology and nitrogen use efficiency in the black-soil rice area of Northeast China. However, the yield response of FM and SA when coupled with different irrigation regimes is far from a simple linear superposition. If mulch is superimposed on top of continuous flooding, the mulch-driven warming and the flooding-driven reducing conditions may jointly create a high-temperature, anoxic inter-root environment that inhibits root function and effective spike formation [9]. Because of this, a systematic field evaluation of the coupled “irrigation system × supporting measures” on albic soil in the Sanjiang Plain is still insufficient.

Conventional ANOVA is designed to test whether treatment means differ, and for treatment comparison, it is entirely appropriate. Objectives (ii) and (iii) of this study, however, concern not group means but structural relationships—identifying the core driver among strongly collinear physiological predictors, and identifying treatments that deviate from the overall yield–physiology relationship rather than merely yielding less. ANOVA represents neither the indirect paths among predictors nor a fitted relationship from which a treatment can depart, so the limitation is one of dimensional mismatch between tool and question rather than of relative performance. In terms of methods for identifying yield drivers, Interpretable Machine Learning (IML), which has emerged in recent years, provides a tool for parsing such multi-factor coupled data that conventional ANOVA-based analysis cannot, by resolving the structural relationships among collinear predictors and by diagnosing treatments that depart from the overall yield–physiology relationship, but the reliability of its conclusions remains controversial. Ryo [10] systematized the methodological framework of IML and Explainable Artificial Intelligence (XAI) in agricultural data analysis, laying the foundation for applications, but subsequent studies continue to reveal the limitations of a single model. Hu et al. [11], after comparing six types of black-box models, pointed out that Random Forest and XGBoost are prone to pseudo-correlation when extrapolating to new climate scenarios, thus misjudging the yield-driving mechanism. Zhang et al. [12] construct a hybrid model with DSSAT and SHapley Additive exPlanations (SHAP) to further demonstrate that the key drivers identified by any single algorithm have limited robustness under different modeling assumptions. This problem is particularly acute in multi-treatment field trials, i.e., the sample sizes of such trials are generally small (n = 27–54), and it is often difficult for a single model’s ordering of feature importance to remain stable across hypotheses. In view of this, instead of relying on a single model, this study integrates multiple modeling approaches and couples conformal prediction with consistency testing to enhance the reliability of mechanism inference under small-sample conditions by cross-validating the consistency of factor contributions under different assumptions.

To address these gaps, a two-year multi-treatment field trial (2024–2025) was conducted on albic soil (Albic Luvisols) in the Sanjiang Plain with three objectives: (i) quantify the effects of nine coupled “irrigation regime × auxiliary practice” treatments on yield, WUE, and yield components, and identify the preferred scheme and the lower CI safety threshold; (ii) identify core physiological yield predictors via a multi-model consensus matrix; and (iii) develop and validate a multilevel diagnostic framework for detecting yield-response outliers under small-sample conditions, supported by companion Monte Carlo simulations. Two hypotheses were tested: H1—SACI achieves three-dimensional synergy of yield, WUE, and water saving through improved root morphology and rhizosphere redox status; H2—FMCG constitutes a yield-response outlier driven by hot–anoxic rhizospheric inhibition.

## 2. Materials and Methods

### 2.1. Test Site and Meteorological Conditions

**Figure 1:**
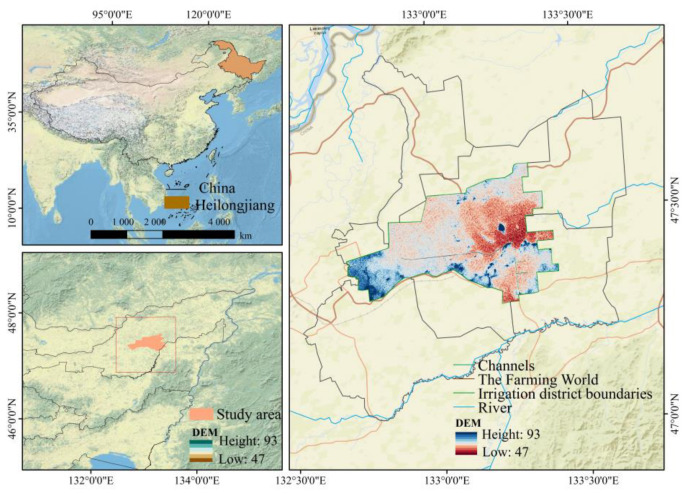
Overview map of the study area.

The field experiment was conducted during the 2024 and 2025 rice-growing seasons at the Qinglongshan National Agricultural Science and Technology Park (47°15′ N, 133°28′ E, 55 m a.s.l.), Sanjiang Plain, Heilongjiang Province, China. The area has a temperate humid continental monsoon climate, with an average annual temperature of 2.5 °C and a frost-free period of 130–140 d. The test plots in the two years were perennial rice fields, and the management of the previous crop was consistent with the basic fertility. The soil at the experimental site is classified as an albic soil (Albic Luvisols, WRB), with the following baseline physicochemical properties in the 0–20 cm tillage layer: field water-holding capacity (θfc) 38.2% (*v*/*v*), bulk density 1.32 g cm^−3^, pH 5.80, soil organic matter 32.4 g kg^−1^, total nitrogen 1.85 g kg^−1^, available phosphorus (Olsen-P) 18.6 mg kg^−1^, and available potassium 142 mg kg^−1^; the coefficients of variation (CV) of each index between treatments were less than 4.5%, indicating that the background homogeneity of the plots was good. Below the albic topsoil, a dense clay-illuvial argic horizon, with clay content markedly higher than the overlying albic layer and low saturated hydraulic conductivity, forms a quasi-impermeable confining layer that underlies the soil’s tendency toward perched-water subsurface waterlogging. Whole-growing-season precipitation in 2024 and 2025 was 387 mm and 412 mm, mean daily air temperature was 19.6 °C and 19.8 °C, and active accumulated temperature reached 2348 °C d and 2372 °C d, respectively (meteorological data from the on-site automated weather station). The meteorological backgrounds of the two years were highly consistent, which provided a basis for the reliable separation of the effects of the treatments (Figure 1).

### 2.2. Experimental Design

A total of nine “irrigation regime × auxiliary practice” coupled treatments (Table 1) were arranged in a randomized complete block design (RCBD) with three replications per year, yielding a cumulative sample size of n = 54 plot-seasons over the two years. Each plot measured 30 m^2^ (5 m × 6 m), surrounded by a 0.5 m non-sampled border strip (protective rows) on all sides to eliminate edge effects; only the inner 4 m × 5 m effective area was used for sampling and yield determination. A vertical PVC waterproof membrane was buried to 40 cm depth along plot ridges to block lateral water seepage between treatments. Within each plot, all destructive sampling positions (tiller quadrats, root auger cores, and yield harvest areas) were determined by computer-generated random coordinates at the start of each season, and sampling locations were marked with permanent stakes to ensure consistent spatial randomization across growth stages and between years. The amount of irrigation water was accurately measured by a separate float-type water meter.

### 2.3. Agronomic Management

The test variety is ‘Longjing 31’, the main cold-region japonica rice variety in Heilongjiang Province, which is one of the high-quality, medium- and early-maturing Japonica rice varieties with the largest popularized area in the rice area of the Sanjiang Plain at present. In both years, seedlings were nursery-sown on 14 April and manually transplanted on 2 June at a spacing of 30 cm × 14 cm (23.8 × 10^4^ hills ha^−1^) with three seedlings per hill; harvest occurred in mid-to-late September, with a field growth period of 108–110 d. The fertilization program remained consistent across treatments: 240 kg ha^−1^ of N (40% basal, 30% tiller, and 30% panicle fertilizer), 90 kg ha^−1^ of P_2_O_5_ full basal, and 120 kg ha^−1^ of K_2_O (60% basal, 40% panicle fertilizer).

SA is a humic acid-based composite amendment, the main components of which are humic acid (≥30%), diatomaceous earth (≥15%) and bentonite (≥20%), with trace elements such as Fe (0.8%), Zn (0.3%) and Mn (0.4%); The soil was spread at 3.0 t ha^−1^ 7 d before transplanting and rototilled to 0–15 cm to mix well. Film Mulching (FM) used 0.008 mm black polyethylene (PE) film, which was covered between rows immediately after transplanting, with a coverage rate of about 65%, and manually recovered 10 d before harvest.

### 2.4. Sampling and Measurement

During the critical reproductive period of rice, this study synchronized the monitoring of indicators at four levels: canopy physiology, root morphology, inter-root microenvironment and yield (Table 2). In this case, the inter-root microenvironmental data was collected separately from the machine learning modeling data stream to serve as independent evidence at the mechanism level, thus avoiding covariance coupling between it and the yield prediction features.

Canopy physiological indices were measured once at the tillering, nodulation and gestation, tasseling and flowering, and milky ripening stages. The net photosynthetic rate (Pn) was measured with the LI-6800 Portable Photosynthesis System (LI-COR Biosciences, Lincoln, NE, USA) in the uppermost fully expanded leaf of the main stem, with leaf compartment settings of PAR = 1200 μmol m^−2^ s^−1^, reference CO_2_ = 400 μ mol mol^−1^ and was performed on a sunny day from 09:00 to 11:30, with five replications per treatment. SPAD values (relative chlorophyll content) were averaged over the upper, middle and lower segments of the inverted bilobed, and 10 slices were measured per treatment. Tiller dynamics were investigated every 5 d from 10 d after transplanting, and five 0.25 m^2^ fixed sample plots were set up in each plot. The aboveground dry matter was sampled from nine holes at each measurement period, separated by organ, dried at 80°C until constant weight and weighed.

Root morphology was determined at a single developmental stage, the milk (grain-filling) stage, which constitutes the decisive window for yield formation in cold-region japonica rice (Section 4.1). Sampling was confined to this stage because the WinRHIZO morphometry described below entails destructive root–soil excavation that cannot be repeated across successive stages without perturbing the soil–water dynamics under investigation. Three randomly selected holes per plot were sampled with a 30 cm × 30 cm × 30 cm root auger and scanned with a WinRHIZO Pro root analysis system (Regent Instruments, Quebec, QC, Canada) to resolve the distribution of total root length, surface area, volume, and root diameter, after being flushed through a 0.5 mm sieve to remove soil impurities. Root samples were dried at 80 °C until constant weight and used to calculate the root-crown ratio.

The inter-root microenvironment consisted of three main determinants. Redox potentials (Eh) were measured in situ in the field to a depth of 5–8 cm using a FJA-6 ORP meter (Shanghai INESA Scientific Instrument Co., Ltd., Shanghai, China) with Pt/SCEs, and the instrument was calibrated with a ZoBell standard solution, with three mean values taken from each test plot. The total reducing power of the root system was determined by a modified TTC (2,3,5-triphenyltetrazolium chloride) method; i.e., 0.5 g of milky-ripened fine roots (<1 mm in diameter) were taken and incubated in the dark at 37 °C for 3 h. The triphenylmethylfouling (TPF) produced was colorimetrically quantified at 485 nm wavelength using a UV-1800 spectrophotometer (Shimadzu Corp., Kyoto, Japan), and the reducing activity of the roots was characterized by the yield of TPF per unit of root mass. Root zone temperatures were automatically recorded by the Echo-T probe (METER Group, Inc., Pullman, WA, USA; accuracy ±0.1 °C) at 5 cm depth every 30 min, and the daily average temperature was calculated during the irrigation period (11:00 to 15:00), and then the temperature difference (ΔT) between the treatments and the conventionally flooded control (CK) was used to characterize the warming effect in the root zone. Dissolved O_2_ concentration was not measured directly; rhizosphere Eh and root TTC-reducing activity were used as established in situ proxies for the rhizosphere redox/oxygen status.

### 2.5. Technical Framework

**Figure 2:**
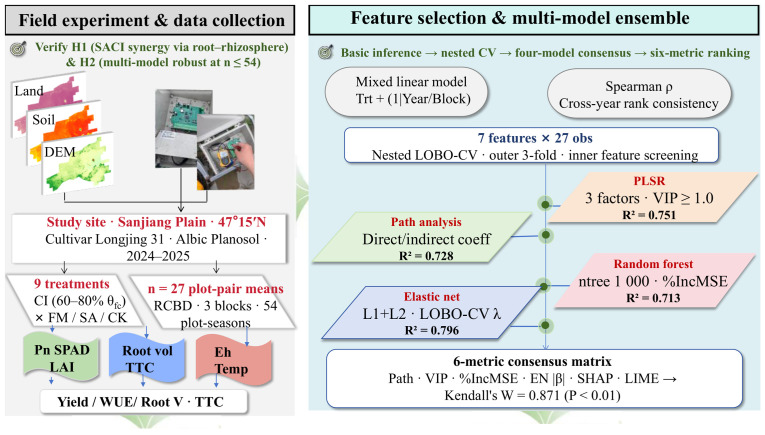

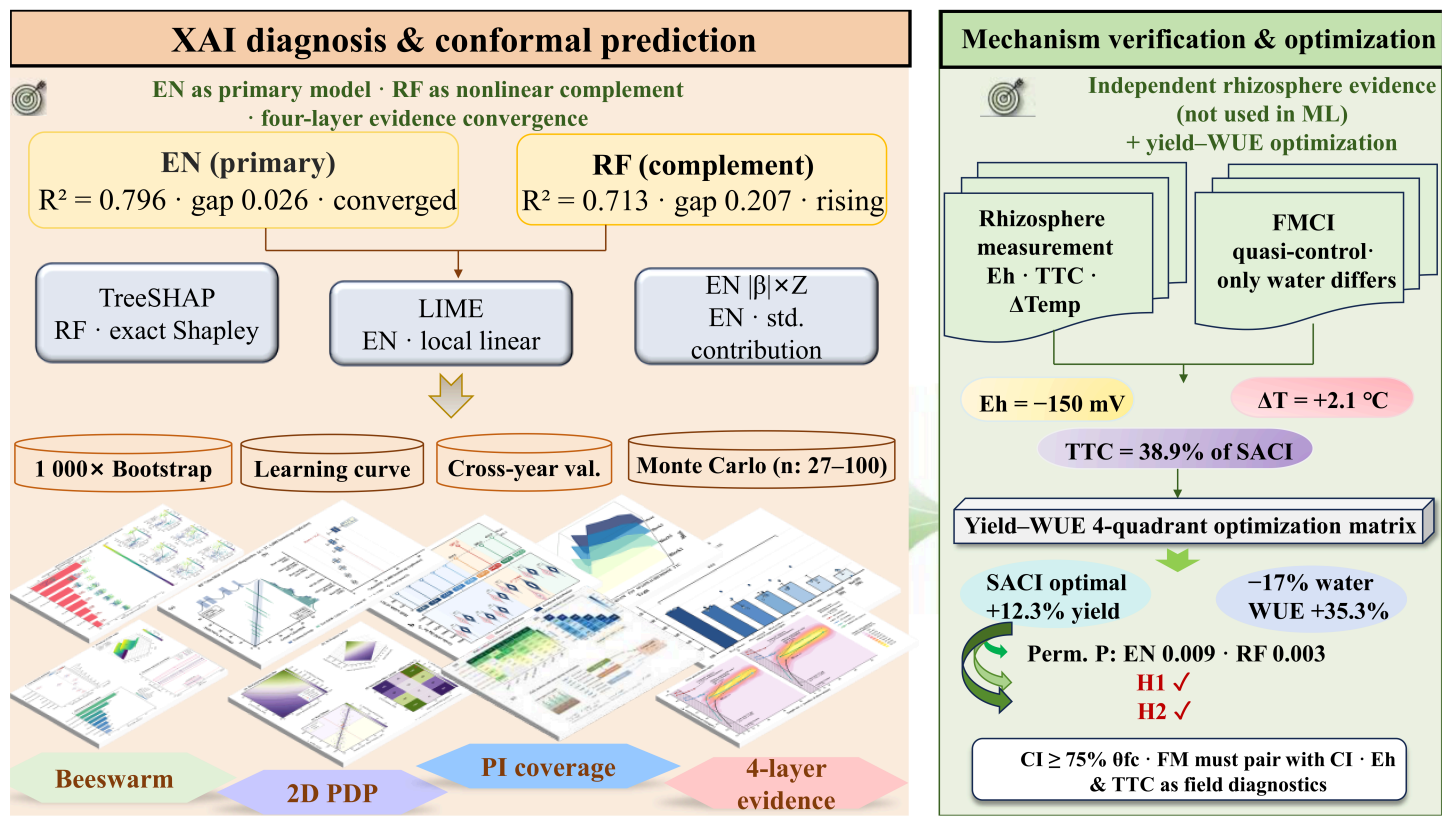
Research framework. Abbreviations: EN, elastic net; RF, random forest; PLSR, partial least squares regression; VIP, variable importance in projection; %IncMSE, percent increase in mean squared error; SHAP, SHapley Additive exPlanations; LIME, Local Interpretable Model-agnostic Explanations; PDP, partial dependence plot; PI, prediction interval; LOBO-CV, leave-one-block-out cross-validation; XAI, explainable artificial intelligence.

The technical framework of this study is illustrated in Figure 2.

### 2.6. Yield Measurement and Water Use Efficiency

Harvesting was carried out at the physiological maturity (18 September 2024 and 20 September 2025) to measure yield in separate plots. In each plot, three 2 m × 2 m (4 m^2^) sample plots were randomly selected and harvested together after setting aside a buffer strip of 0.5 m from the edge of the plot, for a total of 12 m^2^ in the three sample plots. Seeds were threshed by a plot thresher (SBS-500 model, Zhejiang Topunon, Hangzhou, China), dried naturally for 7 d and manually cleared of impurities and then weighed with an electronic balance (accuracy ±0.1 g), moisture content was determined using a PM-8188-A Digital Grain Moisture Meter (Kett Electric, Tokyo, Japan), and yields converted to the international standard of 14% moisture content *Y*_14_ (kg ha^−1^):

(1) $$Y_{14}=W_{field}\times \frac{1-M_{field}}{1-0.14}\times \frac{10000}{12}$$

In the equation: $W_{field}$ represents the measured fresh weight of grains in the plot (kg); $M_{field}$ is the measured moisture content (decimal); 10,000/12 is the area conversion factor. Effective number of panicles (panicles m^−2^, five 0.25 m^2^ fixed sample plots per plot), number of grains in a panicle (30 holes per plot), filled-grain percentage (%) and thousand kernel weight (g, three 1000-grain replicates per plot) were measured simultaneously.

Water use efficiency is defined as production output per unit of water consumed:

(2) $$WUE=Y_{14}/W_{total}$$

In the formula: $W_{total}$ is the total water consumption (m^3^ ha^−1^) during the whole reproductive period, which is accounted for treatment by treatment through the water balance method:

(3) $$W_{total}=W_{irr}+W_{pre}-W_{run}-W_{per}+\Delta W$$

where $W_{irr}$ is the effective volume of irrigation water (read by a separate water meter), $W_{pre}$ is the effective rainfall (converted to rainfall station observations by the 0.7 cut-off coefficient calibrated to rainfall-runoff measurements in this test plot), $W_{run}$ for field surface runoff losses (obtained by weighing runoff collection buckets connected to plot outlets), $W_{per}$ for deep seepage losses (continuous monitoring by self-pressurized seepage meters, recorded hourly), Δ*W* is the difference between the initial and final 0–40 cm soil water storage (measured by drying method at 5 sampling points 1 d before transplanting and the next day of physiological maturity). Water-saving rate was calculated based on CK:

(4) $$Water\;saving(\%)=(W_{total,CK}-W_{total,t})/W_{total,CK}\times 100$$

### 2.7. Statistical Analysis and Modeling Framework

The statistical analysis followed six incremental steps: (1) basic inference, (2) nested feature screening, (3) multi-model integration with interpretable attribution, (4) conformal prediction and permutation testing, (5) robustness diagnosis, and (6) companion simulation validation.

#### 2.7.1. Basic Inference

The effects of treatment and year on grain yield were analyzed using a mixed linear model. Treatment was specified as a fixed effect, while year and within-year block groups were specified as nested random effects. Model parameters were estimated by the restricted maximum likelihood (REML) method, and the denominator degrees of freedom for the fixed-effects F-test were approximated using the Kenward–Roger method. The significance of the year × treatment interaction was evaluated by a likelihood ratio test, and the cross-year consistency of treatment means was assessed by Spearman’s rank correlation coefficient (test statistics reported in Section 3.1).

The unit of analysis for the subsequent machine-learning modeling was defined as the two-year mean of each plot pair (n = 27), rather than as individual plot-seasons (n = 54). This choice was dictated by the statistical-validity requirements of the main inferential framework, which are threefold. First, Jackknife+ conformal prediction guarantees a 1 − 2α coverage lower bound under the exchangeability assumption; however, paired observations from the same plot in two consecutive years carry substantial interannual autocorrelation (quantified in Section 3.1), so treating 54 plot-seasons as independent units would systematically violate this assumption. Second, pooling across years is permissible only when no significant year × treatment interaction is detected, which is verified by the likelihood ratio test reported in Section 3.1. Third, the between-treatment coefficient of variation substantially exceeded the between-year coefficient of variation (Section 3.1), indicating that yield variability was dominated by treatment effects rather than by interannual fluctuations. Together, these conditions justify n = 27 as an appropriate modeling unit that simultaneously satisfies the exchangeability requirement of conformal prediction and supports unbiased estimation of treatment effects.

The normality and homoscedasticity assumptions required for one-way ANOVA were verified by the Shapiro–Wilk test and Levene’s test, respectively (test statistics reported in Section 3.1). On the basis of these assumption checks, multiple comparisons among treatments were performed using the Tukey HSD method (α = 0.05).

#### 2.7.2. Feature Screening Under Nested Cross-Validation

It is very easy to overestimate the prediction performance and distort the feature ranking if feature screening and model evaluation are done simultaneously on the same dataset [13,14]. To avoid this problem, nested cross-validation (Nested CV) was used in this study. The outer layer was leave-one-zone-group cross-validation (LOBO-CV, 3-fold), where one full zone group (9 plots) was set aside for validation at a time and the remaining 18 plots were used for training. The inner layer, on the other hand, independently performs feature screening and hyperparameter tuning within each outer layer training fold, eliminating any leakage of outer layer authentication information. The screening threshold was set at Pearson |r|>0.60 (*p* < 0.05 after Bonferroni correction). The final response feature set consists of the 7 core features that were consistently selected in all 3 outer-layer training trade-offs (selection rate: 100%), ranked by importance as follows: Root volume at milky stage ($X_{1}$), Above-ground dry matter at milk ripening stage (aboveground dry matter, DM, $X_{2}$), Root surface area at milk stage ($X_{3}$), Net photosynthetic rate at tillering stage (Pn, $X_{4}$), SPAD during milk stage ($X_{5}$), Aboveground DM at heading stage ($X_{6}$) and root DM at milky stage ($X_{7}$). Seed yield Y was the response variable. All features are Z-score standardized before modeling, and their mean and standard deviation are only calculated within each outer training fold, and then the outer validation folds are converted accordingly to prevent information leakage.

#### 2.7.3. Multi-Model Integration and Interpretable Attribution

For small samples of data, a single model’s ordering of feature importance is susceptible to its intrinsic mathematical assumptions, resulting in limited robustness of conclusions. In order to obtain robust conclusions that do not depend on specific assumptions, this study constructs four types of models that are independent of each other based on different mathematical principles. First, path analysis was used to decompose the direct and indirect path coefficients of each variable on yield, following a stepwise regression in which only variables with a variance inflation factor (VIF) < 5 were retained. Second, partial least squares regression (PLSR) was applied, with the optimal number of latent variables determined by LOBO-CV and variables retained according to a variable importance in projection (VIP) ≥ 1.0 threshold. Third, random forest (RF) was trained with ntree = 1000, and mtry was tuned by out-of-bag (OOB) error grid search. Fourth, the elastic net (EN) was jointly tuned by 10-fold cross-validation over a two-dimensional grid consisting of α ∈ {0.1, 0.3, 0.5, 0.7, 0.9} (step 0.05) and λ ∈ {0.001, 0.005, 0.01, 0.05, 0.1} (40 logarithmically spaced points). The optimal hyperparameters obtained from these tuning procedures are reported in Section 3.3. In order to ensure the accuracy of the comparison of the predictive performance of each model, the four types of models were uniformly evaluated by LOBO-CV R^2^. The learning curve diagnostic results provide a clear basis for model selection, i.e., the difference between the R^2^ of the RF training set and the validation set is still 0.207 at n = 27, indicating that convergence has not yet occurred. The difference between the two ENs is only 0.026, which has converged. Based on the above results, this study identified EN as the main analytical model and RF as the complementary nonlinear control model for the construction of a two-dimensional partial dependency plot (PDP) incorporating SHAP interaction attribution.

We emphasize that this non-convergence concerns RF’s absolute predictive R^2^ (its train–validation gap), not the stability of its feature ranking or attribution direction, which are the only RF outputs the present analysis uses and which were verified independently: milk-stage root volume remained the top RF predictor across 1000 bootstrap resamples (%IncMSE 42.6%, 95% CI [36.2, 49.1]) and in 86.7% of simulation replicates at n = 27, rising to 98.6% at n = 100 (Section 3.7). Moreover, the central feature-importance conclusion does not rest on RF: among the six consensus methods, the four non-RF methods (path analysis, PLSR-VIP, EN |β|, and EN-based LIME) independently rank root volume first.

Accordingly, RF outputs (SHAP, PDP) were used not as a stand-alone inferential basis but as a nonparametric complement that supplies the nonlinear interaction detection EN cannot represent. They were accepted only where they converged with the converged EN (e.g., the directionally identical FMCG diagnosis, EN −378 vs. RF SHAP −480 kg ha^−1^), and their small-sample reliability was explicitly bounded by the companion simulation (Section 3.7).

Based on the four types of models mentioned above, this study further develops a multi-method interpretability analysis. In this case, the SHAP value is based on the RF model and is calculated using the TreeSHAP exact algorithm [15]. Local Interpretable Model-agnostic Explanations (LIME) performs local attribution based on the EN model and uses the EN-normalized coefficient contribution decomposition (|β| × Z(x)) as an alternative attribution path independent of RF/SHAPAs a result, six categories of metrics, namely, pass-through analysis, PLSR-VIP, RF %IncMSE, EN |β|, SHAP |mean| and LIME |mean|, together form a multi-method consensus matrix of feature importance, and the consistency among methods is evaluated by Kendall’s concordance coefficient (W) combined with the two-by-two Spearman ρ. It should be noted that all six methods work on the same training set, and their “consensus” reflects the robustness of the feature importance results under different modeling assumptions and attribution paths, rather than the cumulative evidence from multiple independent tests in the traditional sense. Therefore, Kendall’s W = 0.871 should be interpreted as a measure of methodological robustness rather than a Bonferroni-corrected multiple testing statistic. In addition, this study further implemented a two-dimensional partial dependency plot (PDP) analysis on the RF model to quantify the nonlinear joint effect of key feature pairs (root volume at milking stage versus dry matter above ground at milking stage) on yield and their superadditive increments.

#### 2.7.4. Conformal Prediction and Alignment Testing

Yield uncertainty at the treatment level is quantified by a three-level conformal prediction architecture. The first layer is the global Jackknife+ conformal prediction, which serves as the main analytical layer and follows the theoretical framework of Barber et al. [16] by constructing 95% prediction intervals for EN and RF, respectively, with all n = 27 observations, and the two models give directionally consistent coverage diagnostics, which can be used to form a cross-model corroboration of outlier responses to a single treatment. The second stratum was grouped using Mondrian Conformal Prediction, which served as an auxiliary exploratory diagnostic stratum, clustering the samples by type of companion measure into an unassisted group (CK + CI1–CI4, m = 15), an overlay group (FMCI + FMCG, m = 6) versus a modifier group (SACI + SACG, m = 6). Given the small calibration samples in the latter two groups (m = 6), this stratum serves only the diagnosis of residual heterogeneity and does not advocate strict nominal coverage, which is precisely in line with the downward bias that can be expected from conformal prediction coverage with small samples [17]. The third layer is the residual consistency test, which serves as a distribution-independent statistical layer to find the exact *p*-value of the treatment-level outlier response by randomly permuting the treatment labels 10,000 times and counting the |maximum| of the residual means of the LOBO-CV residuals for each treatment in the subset of the permutation distribution. Replacement seeds were fixed (SEED = 20,240,618) to ensure reproducible results.

#### 2.7.5. Robustness Diagnosis

The robustness of the model was assessed using a combination of the following five diagnostics. i. Conduct 1000 bootstrap resamples to estimate 95 per cent empirical confidence intervals for the model significance indicators. ii. Learning curve diagnostics, i.e., the sample size n was incremented from 9 to 27 in steps of 3, and 100 bootstrap resamplings were repeated at each value of n to examine the convergence of the model and the bias-variance trade-offs. iii. Conduct generalized validation across years, training with 2024 data, testing with 2025 data and repeating the process in reverse. iv. Calculate Spearman’s rank correlation coefficient ρ for the feature ordering under both LOBO-CV and conventional cell-level LOO-CV schemes to assess the consistency between the two. v. Test the cross-fold consistency of feature selection in nested CVs: if a feature is selected in all three outer training folds (100% selection rate), it is recognized as a stable feature.

#### 2.7.6. Supporting Simulation Studies

To further quantitatively examine the bias-variance behavior of the above diagnostic frameworks and their efficacy in detecting outliers in small samples, this study conducted companion simulations. Synthetic data is structured as follows: “inter-feature covariance × actual yield at treatment levels + FMCG-type outlier effects and additive noise”:

(5) $$X∼N(\mu k,\sum X),Yk=X\beta +\alpha k\cdot 1\left\{k=FM\left.CG\right\}\right.+\varepsilon ,\varepsilon ∼N(0,\sigma^{2}\varepsilon )$$

In this equation, the mean vector *μk* and covariance matrix Σ*X* of *X* are estimated from the measured data, and the regression coefficient β is taken from the standardized regression coefficient of EN. It should be noted that, because *X* and *β* are inherited from the observed dataset, the companion simulation is intended to characterize the small-sample statistical behavior of the diagnostic framework, namely its bias-variance properties, finite-sample conformal coverage, permutation-test power, and cross-model agreement, rather than to provide independent external validation of the feature ranking. The latter is established independently of the observed data through the six-method consensus and the model-free rhizosphere evidence and is therefore not contingent on the simulation. Outlier effect intensity: three levels (the measured mean residual for FMCG treatment is approximately −860 kg·hm^−2^). The error variance σ^2^ε is calibrated so that the overall coefficient of determination of the model R^2^ is about 0.78, which is comparable to the LOBO-CV R^2^ of the measured ENs. The sample size gradient is set at three levels of n∈{27,50,100}, corresponding to the actual observed sample size, medium expansion and large sample limit, respectively; Each ($n,\alpha_{k}$) combination was independently replicated 1000 times (pseudo-randomized seeds in order 1–1000). The simulations assessed the stability of the core feature ordering as measured by the frequency of the first root volume at milky maturity and the average Spearman ρ of the top three feature orderings; Variance-bias decomposition for the EN and RF models; empirical coverage of Jackknife+; Mondrian’s actual coverage rates for the three stratification levels m = 3, 6, and 15; Efficacy of Ranked Tests for Testing Different Outlier Strengths; and Trigger Frequency for Cross-Model Consistency Diagnostics between EN and RF.

### 2.8. A Four-Level Evidence Framework for Outlier Processing Diagnosis

Machine learning’s single diagnostic conclusion for small sample data may produce epistemological artifacts due to limitations in the interpretation methodology, model family induction bias, and random sample perturbations, and reliance on a single model’s interpretive results runs the risk of being misled by endogenous uncertainty in the “black box” [11]. To this end, this study constructs a four-layered progressive evidence convergence framework to cross-validate the aforementioned multi-model integration, multi-method interpretable attribution and conformal prediction results to identify yield response outlier treatments. Each of the four layers of evidence is designed to address four different sources of epistemic risk in a single diagnosis, complementing but not implying each other.

(i) Layer 1 Multi-perspective self-consistency: RF-based SHAP attribution, EN-based LIME attribution, LOBO-CV residuals, and Jackknife+ conformal interval coverage should corroborate one another across the two analytical models. This layer guards against methodological artifacts of a single explanatory approach, such as the instability of SHAP attribution to correlated features or the proxy error of LIME local linear approximation to nonlinear response surfaces. When the four categories of same-model evidence diverge, they need to be traced back to the plausibility of the original model fit. This layer is necessary for outlier response identification, but conclusions relying only on this layer suffer from model selection dependence.

(ii) Layer 2 EN and RF cross-model consistency: EN (linear + L1/L2 regularization) and RF (nonparametric integration tree) represent two completely different types of inductive bias between parametric linear and nonparametric nonlinear assumptions, respectively, which are not dependent on each other in terms of mathematical structure, handling of feature interactions and sensitivity to noise. If a diagnosis with consistent direction is given simultaneously for the same treatment (residuals of the same sign, simultaneous failure of coverage of conformal intervals, and consistent first-place negative contributing features), the conclusion is independent of the choice of the specific model family and constitutes the key independent evidence of model-family agnosticism (model-class agnostic). This layer guards against the possible distortion of conclusions by model-family generalization bias.

(iii) Tier 3 Mechanistic-Level Independent Evidence: Three independent physicochemical measurements of rhizosphere Eh, TTC reducing power and rhizosphere temperature (which are not involved in any of the ML modeling), and a quasi-experimental control between the FMCI and the FMCG (which share mulch treatments and differ only in water management practices) together provide independent pathways outside the model. This layer addresses the inherent epistemological limitation of machine learning, i.e., that any ML model can only reveal statistical correlations, not confirm physiological mechanisms [11], and thus independent evidence at the mechanism level is a necessary bridge to elevate “statistically significant outliers” to “mechanistic outliers”.

(iv) Layer 4 Distribution-independent permutation test for significance: As a robust bridge between the statistical and mechanistic layers, the residual permutation test in the previous section gives the exact *p*-value of the treatment level outlier response without assuming the distribution of the residuals, and without relying on the accuracy of the model parameter estimation. This layer guards against potential violations of distributional assumptions in parametric inference and provides probabilistic bounds in a frequentist sense for the “convergence of evidence” observed in the first three layers.

In summary, the four layers of evidence are epistemologically independent and complementary to each other: the first layer protects against artifacts of the explanatory method, the second layer protects against inductive bias of the model family, the third layer bridges the gap between statistics and mechanism, and the fourth layer protects against distributional assumption violations. Outlier response identification has the highest confidence only when all four layers of evidence above converge to point to the same treatment; this convergence logic simultaneously constitutes multiple independent tests of the probability of a false-positive outlier response for that treatment. The actual reliability bounds for each tier in the small sample (n = 27) condition were quantitatively tested by the accompanying simulations.

## 3. Results

### 3.1. Overall Treatment Effects: Yield, Yield Components and Water Use Efficiency

Mixed linear modeling analysis showed that the nine irrigation methods coupled with the auxiliary measure treatments differed significantly in seed yield on a total of 54 plot-seasons over the two years (F_8,40_ = 28.6, *p* < 0.001, denominator degrees of freedom as approximated by the Kenward -Roger approximation; Table 3), with yields ranging from 8976.0 to 10,578.2 kg ha^−1^ between treatments.

The year × treatment interaction effect was not significant (likelihood ratio test $\chi^{2}=6.42,df=8,P=0.601$). The coefficient of variation between treatments (CV = 8.4%) was much higher than that between years (CV < 1.2%), which indicated that the yield variability was mainly due to treatment effects. This result provides a statistical basis for the subsequent use of the same plot mean (n = 27) in both years as the unit of analysis for machine learning.

Furthermore, the Spearman rank correlation between treatment means of the two years reached ρ = 0.967 (*p* < 0.001), confirming a high interannual consistency of treatment ordering and thereby supporting the use of two-year plot-pair means as the analytical unit (n = 27) in the subsequent machine-learning modeling. The Shapiro–Wilk normality test (W = 0.978, *p* = 0.412) and Levene’s homoscedasticity test (*p* > 0.05) further indicated that the data satisfied the prerequisites for one-way ANOVA, justifying the use of the Tukey HSD procedure for the multiple comparisons reported in Table 3.

**Figure 3:**
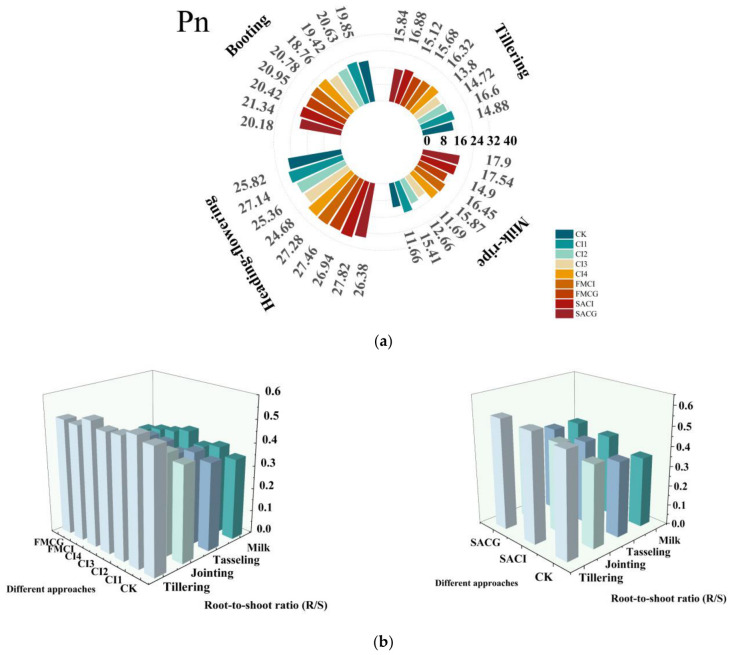

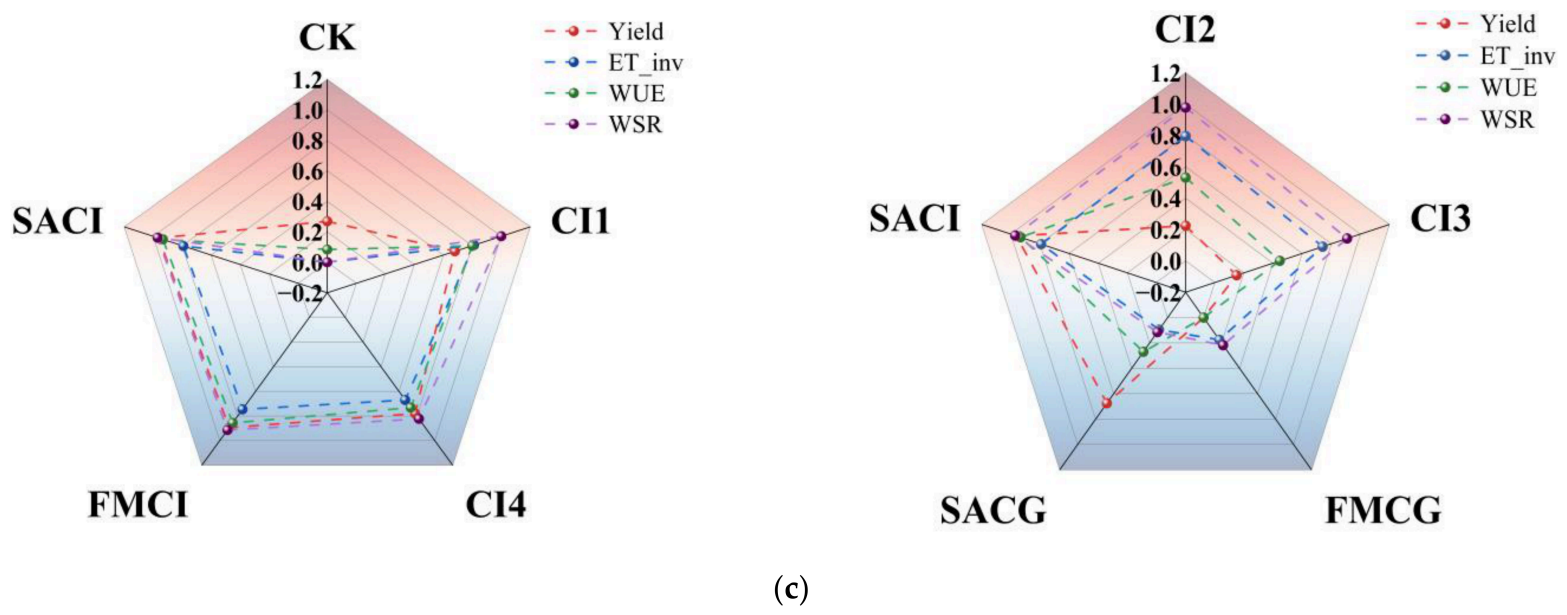
Whole-season canopy, root, and water-use performance of rice under the nine coupled “irrigation × auxiliary practice” treatments. (**a**) Dynamics of net photosynthetic rate (Pn, μmol CO_2_ m^−2^ s^−1^) of the second-from-top leaf across four growth stages (peak tillering, jointing–booting, heading–flowering, milk-ripening). (**b**) Dynamics of root-to-shoot ratio (R/S) across the same four growth stages. (**c**) Radar plot of normalized grain yield, inverse evapotranspiration (ET_inv_), WUE, and water-saving rate (WSR) across all nine treatments; all four indicators were min–max normalized, with SACI consistently occupying the outermost vertices, indicating tri-dimensional synergy among yield, WUE, and water saving. Treatment codes are defined in Table 1; error bars indicate ± SE (n = 3).

The soil-amendment-coupled 75% θfc CI treatment (SACI) exhibited a three-dimensional synergistic advantage: a 12.3% yield increment (*p* < 0.05), a 17.0% water saving, and a WUE of 1.801 kg m^−3^—35.3% higher than CK (Figure 3c). Tukey HSD pairwise comparisons (Table 3) further showed that SACI yield was significantly higher than that of seven of the eight remaining treatments (*p* < 0.05); the sole exception was FMCI (10,464.9 kg ha^−1^, letter group “ab”, *p* > 0.05), which shared the letter “a” with SACI. Nevertheless, SACI attained the highest WUE (1.801 vs. 1.761 kg m^−3^) and the lowest total water consumption among all CI-based treatments (5872.5 vs. 5942.6 m^3^ ha^−1^), and its Jackknife+ prediction interval was the narrowest among all nine treatments (Section 3.6), supporting its designation as the preferred coupling strategy on the basis of three-dimensional synergy. Importantly, this advantage was not driven by a single favorable year: SACI ranked first in both grain yield and WUE in each individual year, and within-year Tukey HSD comparisons independently confirmed the SACI–CK yield difference at *p* < 0.05 for both 2024 and 2025. The non-significant year × treatment interaction (*p* = 0.601) and the near-perfect inter-year rank correlation (ρ = 0.967) further indicate that the reported two-year means faithfully represent a reproducible treatment effect. Comparison between CI gradients showed that 75% θfc (CI4) and 80% θfc (CI1) maintained yields above 10,000 kg ha^−1^, whereas 60% θfc (CI3) had lower yields than CK due to excessive water deficit. The marginal contributions of SACI and FMCI were further disaggregated using CI4 as the baseline: SACI increased yield by 304.6 kg ha^−1^ (+2.96%, *p* < 0.05), and FMCI increased yield by 191.3 kg ha^−1^ (+1.86%, not significant) over CI4. Of the 12.3% total yield increase in SACI over CK, CI by itself contributed 9.3 percentage points, and the marginal contribution of soil conditioner based on 75% θfc CI was 3.0 percentage points.

**Figure 4:**
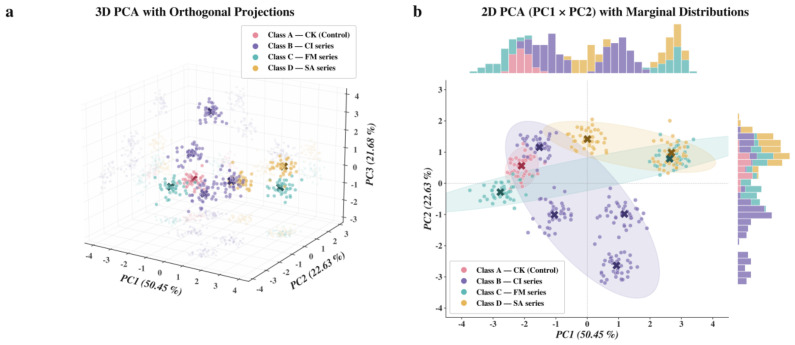
Principal component analysis (PCA) of the multivariate plot-level profile (n = 27, two-year means of seven retained predictors used in subsequent ML modeling) demonstrating cluster separation among treatment classes. (**a**) 3D PCA score plot with orthogonal projections onto the PC1–PC2, PC1–PC3, and PC2–PC3 planes; cumulative variance explained by PC1–PC3 = 94.77%. (**b**) 2D PCA score plot (PC1 × PC2) with marginal kernel-density distributions for each class. Class A = CK (continuous flooding control); Class B = CI series (CI1–CI4); Class C = FM series (FMCI, FMCG); Class D = SA series (SACI, SACG). Colored ellipses denote 95% confidence regions; cluster centroids are marked with star symbols.

Principal component analysis of the seven retained variables (n = 27 plot means) showed that PC1–PC3 cumulatively explained 94.77% of the variance (50.45%, 22.63%, and 21.69%, respectively; Figure 4), confirming clear multivariate structure among treatments. The nine treatments formed four clusters in the subspace formed by the first three principal components: CK formed a cluster independently (Class A); CI1 to CI4 formed a continuous gradient cluster along the direction of the principal components (Class B); the mulch series FMCI and FMCG were located together on the side of the high value of PC1 to form a Class C, but the two were significantly separated on PC2; The soil-amendment treatments (SACI and SACG) together formed Class D. Notably, FMCG diverged markedly from FMCI and the rest of the Class C cluster along PC2, exhibiting characteristics of a potential multivariate outlier. This initial observation of clustering at the multivariate level will be subjected to more rigorous statistical validation through a four-tier evidence aggregation framework in Section 3.5.

**Figure 5:**
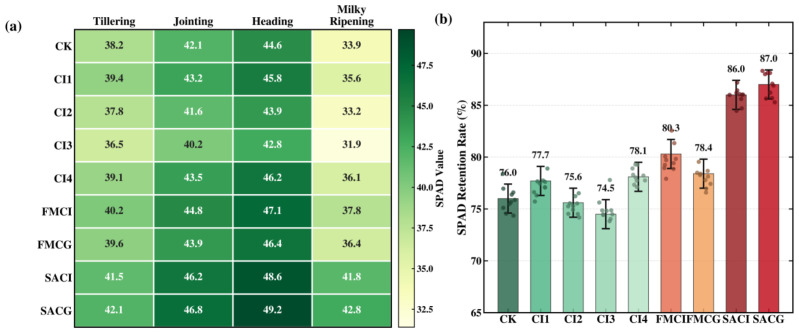
SPAD value dynamics and milk-stage retention rate across the nine treatments. (**a**) Heatmap of SPAD values at four growth stages (tillering, jointing, heading, milk-ripening) for each treatment. (**b**) Ranked bar plot of milk-stage SPAD retention rate (SPAD milk/SPAD heading × 100%). Error bars indicate ± SE (n = 3); treatment codes are defined in Table 3. The strong association between milk-stage SPAD retention and grain yield (r = 0.78, *p* < 0.001) indicates that delayed leaf senescence sustains source capacity through grain filling, whereas the low retention of FMCG and CI3 reflects an early source-end decline consistent with their reduced filled-grain percentage.

The overall trend of canopy physiological indexes was consistent with yield (Figure 3a). The net photosynthetic rate (Pn) was highest in SACG and SACI at the milky stage, reaching 17.90 and 17.54 μmol CO_2_ m^−2^ s^−1^, respectively, which were significantly higher than that of CK (11.66 μmol CO_2_ m^−2^ s^−1^). Strikingly, FMCG presented a counter-intuitive pattern: its P_n at the milk stage (14.90 μmol CO_2_ m^−2^ s^−1^) was significantly higher than those of CI3 (11.69 μmol CO_2_ m^−2^ s^−1^) and CK, yet its final grain yield was the lowest among the nine treatments. This dissociation indicates that instantaneous photosynthetic capacity at a single time point does not linearly translate into season-long assimilate accumulation. SPAD retention at milk maturity relative to heading stage (Figure 5b) was strongly correlated with yield (r = 0.78, *p* < 0.001), with SACI (87.0%) and FMCI (80.3%) significantly higher than FMCG (78.4%) and CI3 (74.5%).

**Figure 6:**
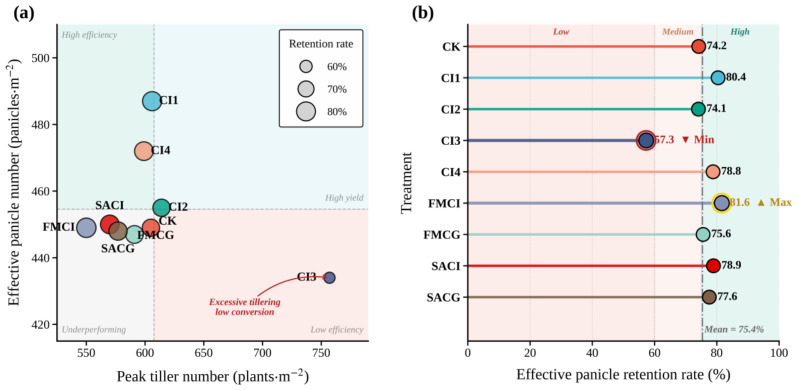
Tiller dynamics and effective panicle retention across the nine treatments. (**a**) Bubble plot of peak tiller number (x-axis) versus effective panicle number (y-axis); bubble size denotes retention rate; quadrant lines delimit the high-yield and high-efficiency regions, with CI3 falling into the “excessive tillering, low conversion” region. (**b**) Effective panicle retention rate (effective panicles/peak tillers × 100%) ranked across treatments; “Min” and “Max” markers identify extremes. Treatment codes are defined in Table 3; n = 5 quadrats (0.25 m^2^ each) per plot.

In terms of yield components, the number of grains in a panicle (97.4 vs. 96.8 grains) and the filled-grain percentage (90.3% vs. 89.6%) were significantly higher in FMCI and SACI than in FMCG (91.4 grains, 84.1%) and CI3 (92.8 grains, 84.6%) (Table 3). Observations on tiller dynamics showed that the CI3 treatment had a typical excess of ineffective tillers: the peak tiller density reached 757 tillers m^−2^, 158 tillers m^−2^ higher than that of CI4 (599 tillers m^−2^), but the effective panicle retention rate was only 57.3% (Figure 6). CI3 fruiting rate (84.6%) was significantly lower than that of CI4 (86.9%), which was consistent with the decreasing trend of effective panicle retention. This result is in line with the direction of existing reports that excessive water deficit significantly reduces the effective tillering rate of rice [18], and the underlying mechanism may involve the suppression of cytokinin synthesis in axillary buds and the disturbance of antagonistic balance between cytokinin synthesis and monocotyledonolactone in the axillary buds under water stress [19,20], which partially rescues the inhibition of the growth of the lower axillary buds and promotes the occurrence of large numbers of ineffective tillers at the later stage of the planting process.

### 3.2. Coupled Response of Root Morphology and Rhizosphere Microenvironment

Root morphological parameters differed significantly among treatments at milky stage (Table S1). SACI root volume amounted to 80.81 cm^3^ hill^−1^, which was 121.5% higher than that of CK (36.48 cm^3^ hill^−1^). The order of root surface area was SACI > SACG > CI1 > CI4 > FMCI ≈ FMCG > CK > CI3, which was consistent with the order of yield. Notably, total root length (802.5 cm hill^−1^), root surface area (796.3 cm^2^ hill^−1^), and root volume (57.31 cm^3^ hill^−1^) of FMCG fell within the middle—rather than the lowest—range of the nine treatments, in striking contrast to its lowest final yield. This dissociation indicates that root morphology alone cannot explain the yield collapse of FMCG. Root-to-crown ratio (R/S, Figure 3b) generally showed a decreasing trend from the jointing–booting stage to the milky stage; SACI maintained a high R/S (0.142) at the milky stage, which was significantly higher than that of FMCG (0.119) and CK (0.127).

The measured data on the rhizosphere microenvironment revealed the functional inactivation mechanism behind the contrast between the morphologically intact and significantly low yield of the FMCG root system (Table 4). The rhizosphere redox potential (Eh) of FMCG was −150 mV, which was at a strongly reducing level, while the rhizosphere Eh of SACI was +80 mV, which was in the mildly oxidizing to neutral range; the difference of 230 mV was the largest inter-treatment difference observed in this study. The direction of this difference is highly consistent with existing water-saving irrigation studies: alternating wet and dry irrigation (AWD) can significantly enhance root oxidative activity and deep root distribution by elevating rhizosphere dissolved oxygen concentration and Eh [21,22]. The TTC reducing power of FMCG roots (168 μg g^−1^ h^−1^) was only 38.9% of that of SACI (432 μg g^−1^ h^−1^) and was also significantly lower than the mean value of the nine treatments (315 μg g^−1^ h^−1^). The average value of the nine treatments (315 μg g^−1^ h^−1^) was also significantly lower. Concomitantly, the rhizosphere temperature of FMCG was 2.1 °C above that of CK, the highest temperature elevation observed among the nine treatments. Collectively, the three independent rhizosphere indicators reveal that FMCG developed a hot–anoxic rhizospheric microenvironment under the coupled effect of mulch-induced warming and prolonged flooding, which resulted in a significant decline in metabolic activity even though the root system remained morphologically intact. The reduction in rhizosphere Fe^3+^ to Fe^2+^ and the accompanying accumulation of reducing phytotoxins under prolonged anaerobic high-temperature conditions have been shown to inhibit root oxidative activity and impair root uptake [23]. The mechanism tier of evidence described above will be used as a third tier of independent evidence to provide support for the outlier treatment diagnosis in Section 3.5.

### 3.3. Model Convergence Diagnosis and Feature Importance Multi-Method Consensus

**Figure 7:**
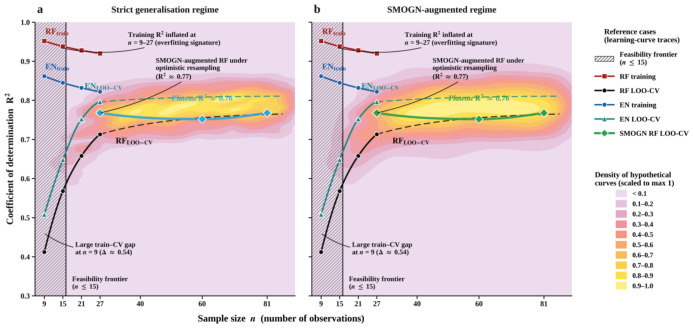
Learning curves of random forest (RF) and elastic net (EN) under leave-one-block-out cross-validation (LOBO-CV). In both panels, solid lines represent the training and LOBO-CV trajectories; the color density represents the distribution of 100 bootstrap-resampled curves per sample size; the dashed horizontal line marks the plateau LOBO-CV R^2^ (≈0.78); and the hatched region denotes the feasibility frontier (n ≤ 15) below which neither model is stable. (**a**) Strict generalisation regime (no synthetic oversampling): EN converges by n ≈ 27 (train–CV gap ≈ 0.026), whereas RF continues to improve up to n ≈ 50. (**b**) SMOGN-augmented regime (training set rebalanced by SMOGN oversampling), shown to confirm that the relative convergence behaviour of EN versus RF is robust to resampling. Together the two panels support the choice of EN as the primary analysis model and RF as the non-linear complement.

All four classes of machine learning models obtain acceptable prediction performance under n = 27, leave-one-block cross-validation (LOBO-CV) conditions (Table S2). The elastic network (EN) model with LOBO-CV R^2^ = 0.796 and the difference between training and validation R^2^ is only 0.026, and the learning curve has reached the plateau; the random forest (RF) model with LOBO-CV R^2^ = 0.713 and the difference between training and validation R^2^ is 0.207, and the learning curve still shows an upward trend at n = 27 (Figure 7); the partial least square regression (PLSR) and the pass-through analysis with LOBO-CV R^2^ are 0.751 and 0.728, respectively.

The hyperparameter tuning procedures described in Section 2.7.3 yielded the following optimal configurations: the optimal number of latent variables for PLSR was 3; the optimal mtry for RF was 3; and the optimal combination for EN was α = 0.5, λ = 0.032.

**Figure 8:**
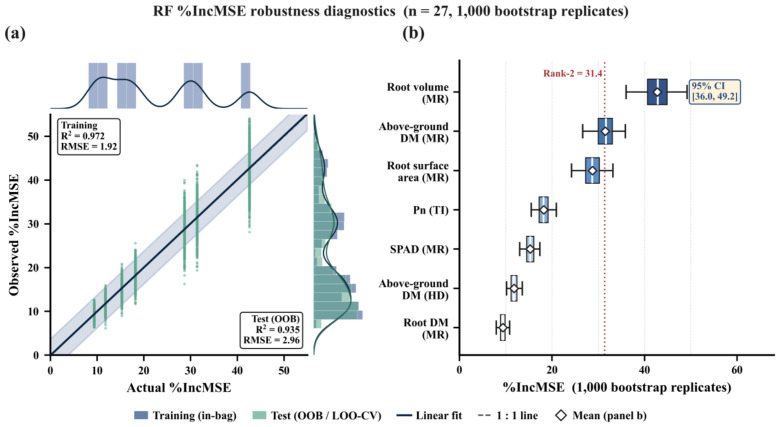
Robustness diagnostics of RF %IncMSE under 1000 bootstrap replicates (n = 27). (**a**) Training-set versus test-set (OOB/LOO-CV) %IncMSE scatter with marginal density distributions; the 1:1 line is shown for reference. (**b**) Bootstrap distribution of %IncMSE for the seven retained predictors (box-and-whisker with diamond means); whiskers represent the 95% empirical confidence interval; the red dashed line marks the rank-2 mean (31.4%). The %IncMSE of milk-stage root volume (top row) is significantly higher than that of any other predictor. Abbreviations: RF, random forest; %IncMSE, percent increase in mean squared error; OOB, out-of-bag; LOO-CV, leave-one-out cross-validation.

Based on the above convergence diagnostics, EN was selected as the main analytical model, and RF was used as a nonlinear complementary control for the two-dimensional partial dependency plot with SHAP cross-correlation. The mean value of %IncMSE for root volume at milk maturity was 42.6% with 95% empirical confidence interval [36.2%, 49.1%] (Figure 8b) under 1000 bootstrap resampling, and the lower bound was significantly higher than the mean value of the second trait, indicating that the root volume was highly robust as the first predictor of the ordination under small sample conditions [11,16].

**Figure 9:**
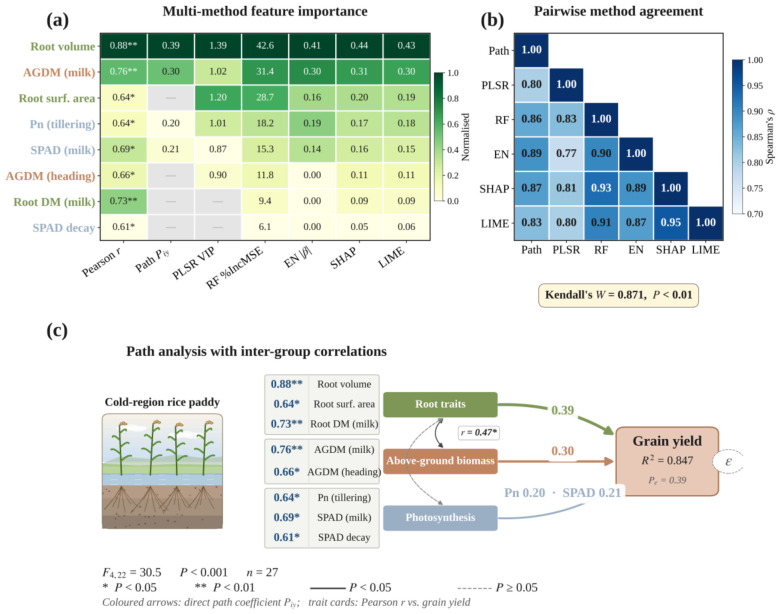
Multi-method feature-importance consensus and path analysis. (**a**) Heatmap of normalized importance from six independent methods (Pearson r, path coefficient, PLSR-VIP, RF %IncMSE, EN |β|, SHAP |mean|, LIME |mean|) for the seven retained predictors at the milk-ripening stage; significance levels are indicated by asterisks (* *p* < 0.05; ** *p* < 0.01). (**b**) Pairwise method agreement matrix based on Spearman’s ρ across the seven feature ranks; Kendall’s W = 0.871 (*p* < 0.01) is shown at the bottom. (**c**) Path diagram showing direct path coefficients (colored arrows; PYi) from root traits, above-ground biomass, and photosynthesis indicators to grain yield, together with trait-card Pearson r against yield; F_4,22_ = 30.5, *p* < 0.001, R^2^ = 0.847, residual ε. Abbreviations: EN, elastic net; RF, random forest; PLSR, partial least squares regression; VIP, variable importance in projection; %IncMSE, percent increase in mean squared error; SHAP, SHapley Additive exPlanations; LIME, Local Interpretable Model-agnostic Explanations. The agreement across the six methods (Kendall’s W = 0.871), together with the indirect path of aboveground biomass routed through root volume (0.182, 37.8% of its total effect on yield), is consistent with root function acting as a central node in the source–sink–transport chain rather than as an isolated predictor.

The Kendall concordance coefficient W = 0.871 (χ^2^ = 36.58, df = 7, *p* < 0.01; Table 5, Figure 9a,b) for the ranking of the 7 core features by the 6 independently interpretable methods (path analysis, PLSR-VIP, RF %IncMSE, EN |β|, SHAP |mean |, LIME |mean |), reached a high level of agreement. All six methods ranked root volume at milky ripening as the most strongly associated statistical predictor of yield—a consensus that reflects predictive robustness under diverse modeling assumptions rather than established physiological causation; five methods ranked dry matter (DM) above ground at milky ripening as the second most strongly associated predictor of yield. PLSR included the root surface area in Priority 2 (VIP = 1.203) due to orthogonality constraints, and the path analysis excluded the DM due to VIF ≥ 5. The two types of methods showed complementary features in terms of covariance treatment strategies. Pearson correlation r = 0.881 (*p* < 0.001) and partial correlation r_partial = 0.642 (*p* < 0.01) were found between root volume and seed yield at the milky stage. Critically, this partial correlation controls for the linear effects of all six other retained features, including root surface area, root DM, and aboveground DM, thereby confirming that the predictive association of root volume with yield is not reducible to shared variance with correlated traits. This robustness is further reinforced by the differential treatment of collinear variables across the six ranking methods: path analysis excluded root surface area and root DM because their VIF exceeded 5 (Table 5, superscript ^a^) while retaining root volume (VIF < 5), and elastic-net regularization independently shrunk aboveground DM at heading stage and root DM to zero (Table 5, superscript ^b^) while assigning root volume the largest coefficient (|β| = 0.412). Pathway analysis (Figure 9c) further showed that the indirect pathway coefficient of aboveground DM to yield via root volume was 0.182, which accounted for 37.8% of its total effect on yield, suggesting that the predicted contribution of aboveground biomass to yield was not completely independent, but partially mediated by the functional dimension of the root system [24].

**Figure 10:**
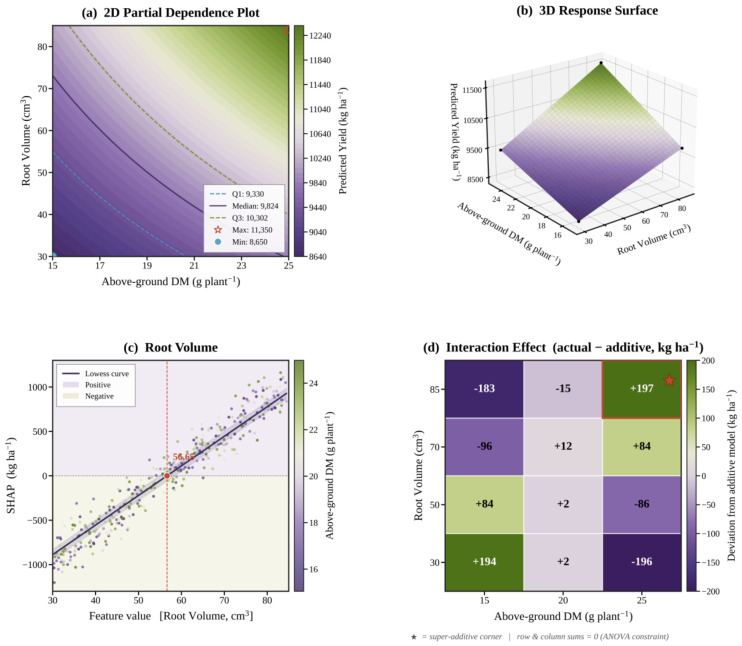
Two-dimensional partial dependence and interaction analysis of milk-stage root volume (RV) and above-ground dry matter (AGBM) on predicted grain yield from the RF model. (**a**) Two-dimensional partial dependence plot (PDP) with predicted-yield isolines (Q1, median, Q3, max, min); the star marks the location of the SACI treatment within the super-additive corner. (**b**) Three-dimensional response surface of predicted yield as a joint function of RV and AGBM. (**c**) SHAP dependence plot for root volume colored by AGBM; the vertical dashed line marks the SHAP turning point (RV ≈ 56.6 cm^3^·hill^−1^). (**d**) Interaction-effect heatmap showing the deviation of the actual joint effect from the additive-model baseline (kg·ha^−1^); the super-additive corner is marked with a star; row and column sums are zero by ANOVA constraint. Abbreviations: RF, random forest; SHAP, SHapley Additive exPlanations. The super-additive corner indicates that high root volume and high aboveground biomass are associated with a greater-than-additive yield gain during grain filling, with the SACI treatment located within this favorable region.

Random forest two-dimensional partial dependence analyses (Figure 10a,b) quantified the nonlinear joint association of root volume and aboveground DM on yield at milk stage: the yield gain when the two were coupled at high values (~2700 kg ha^−1^) exceeded the independent sum (~1930 kg ha^−1^) by ~770 kg ha^−1^, showing a statistically significant superadditivity (Figure 10d). The SHAP dependency plot (Figure 10c) shows a root volume of about 56.6 cm^3^ hill^−1^ as the turning point for positive and negative contributions. The synergistic enhancement of root volume up to 80.81 cm^3^ hill^−1^ (121.5% higher than that of CK) and aboveground DM 32.84 g hill^−1^ (23.7% higher than that of CK) at milking stage of SACI treatment fell exactly in the high yield gain interval of this nonlinear correlation model (Figure 10a star mark in the upper right corner), which is in the same direction of the reported advantage of coupling high biomass and high root function during the filling period in water-saving rice cropping systems [21,25].

### 3.4. SHAP and LIME Two-Path Explanatory Analyses: Global Attribution Patterns and Treatment-Specific Contributions

To further test whether the core feature orderings identified by the multi-method consensus in Section 3.3 are equally robust at the sample-level imputation level, this study employs two mathematically independent imputation paths, the TreeSHAP exact algorithm based on the RF model and the LIME local linear approximation based on the EN model, respectively. Both types of methods provide local-level decompositions of feature contributions for a sample of 27 observations, thereby revealing patterns of treatment-specific attribution outside of the group consensus and providing fine-grained evidence of within-model, multi-perspective self-consistency for the FMCG outlier treatment diagnostics in Section 3.5.

**Figure 11:**
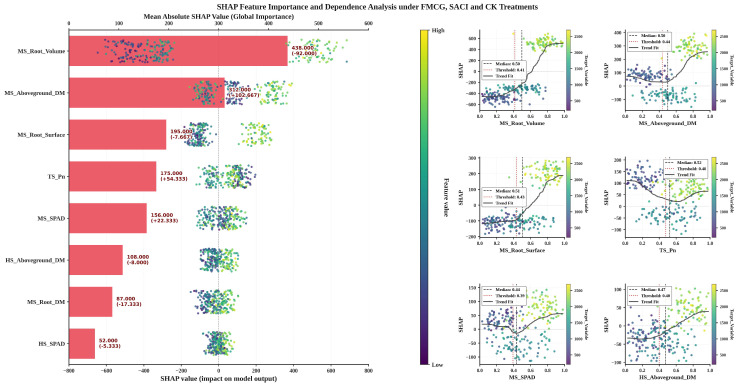
SHAP beeswarm and dependence analysis for the RF model across all 27 plot-mean observations. The bar chart (**left**) ranks the seven retained predictors by mean |SHAP| value (global importance), with milk-stage root volume (MS Root Volume) dominant at 438 kg·ha^−1^—approximately 2.83× the rank-2 feature (above-ground DM at milk stage, MS Aboveground DM, 155 kg·ha^−1^). The beeswarm panels (center) show the per-sample SHAP value distribution for each feature, colored by the standardized feature value (blue = low, yellow = high), revealing directional symmetry whereby high root-volume samples (SACI, FMCI, SACG) cluster on the positive side and low root-volume samples (FMCG, CI3, CK) cluster on the negative side. The dependence panels (**right**) show non-linear SHAP–feature value relationships with LOWESS smoothing; vertical dashed lines mark inflection thresholds (RV ≈ 56.6 cm^3^·hill^−1^; RSA ≈ 530 cm^2^·hill^−1^; SPAD ≈ 36). Abbreviations: SHAP, SHapley Additive exPlanations; RF, random forest; MS, milk stage; TS, tillering stage; HS, heading stage.

The SHAP global imputation result (Figure 11) showed that the root volume at milk stage had the largest |SHAP| mean (438 kg ha^−1^), which was about 2.83 times that of the 2nd aboveground DM (155 kg ha^−1^), which quantitatively corroborated the path-analytical conclusion that root function partially mediates the yield contribution of aboveground biomass. The positive and negative contributions in the swarm plot showed obvious directional symmetry: the SHAP values of the high-root-volume samples (yellow end of the eigenvalues, mostly corresponding to SACI, FMCI, SACG) were concentrated in the range of +300 to +500 kg ha^−1^, while those of low-root-volume samples (blue end of the feature-value spectrum; predominantly FMCG, CI3, CK) clustered between −300 and −500 kg ha^−1^. The SHAP dependency plot further confirmed that the key features of root volume, root surface area and SPAD showed a nonlinear contribution pattern, with turning thresholds at RV ≈ 56.6 cm^3^ hill^−1^, RSA ≈ 530 cm^2^ hill^−1^, and SPAD ≈ 36, in close agreement with the partial-dependence patterns shown in Figure 10c. SPAD and aboveground DM showed a color gradient in the middle and high value regions, suggesting a mild interaction effect between the two. The key information revealed by this swarm diagram is that it not only identifies which variables are important, but also explicitly quantifies in which value interval each variable plays a positive or negative role, constituting fine-grained evidence for mechanism inference [26,27].

**Figure 12:**
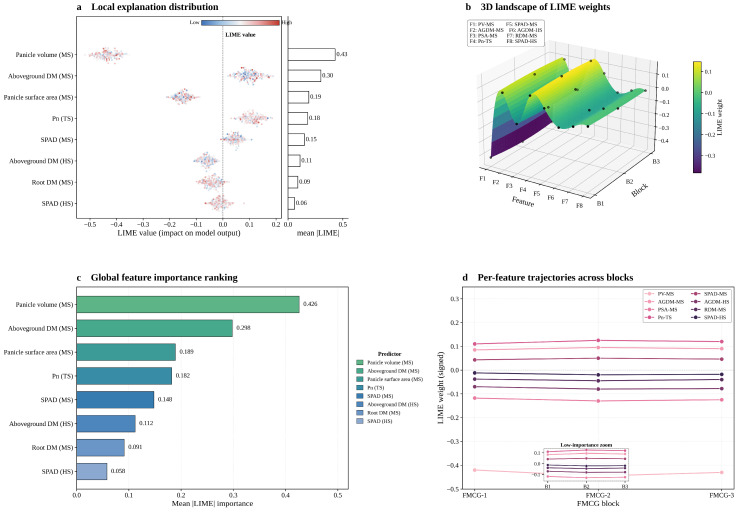
LIME-based local interpretability analysis for the EN model, with FMCG-specific attribution and global ranking. (**a**) Local explanation distribution: per-sample LIME values for the seven retained predictors, colored by feature value (red = high, blue = low); horizontal bars on the right show |LIME| means. (**b**) Three-dimensional landscape of LIME weights across features (F1–F8) and plot blocks (B1–B9), highlighting that milk-stage root volume produces the largest cross-treatment weight contrast (Δ ≈ 0.85). (**c**) Global feature-importance ranking based on mean |LIME| across all 27 observations: root volume (MS) 0.426 > above-ground DM (MS) 0.298 > root surface area (MS) 0.189; this ordering is identical to the SHAP ranking in Figure 11 (Spearman ρ = 0.95). (**d**) Per-feature LIME-weight trajectories across the three FMCG blocks, demonstrating high within-treatment reproducibility (SE < 0.015 for root volume) and confirming that the FMCG attribution pattern is not driven by a single anomalous replicate. Abbreviations: LIME, Local Interpretable Model-agnostic Explanations; EN, elastic net.

The LIME local imputation results (Figure 12) provided imputation pathways that were completely independent of SHAP. The full-sample |LIME| mean rankings based on the EN model (Figure 12c) ranked root volume (0.426), aboveground DM (0.298), and root surface area (0.189) in order of the top 3 at milky ripening, which was identical to the SHAP rankings (Figure 11), with a Spearman ρ = 0.95 (*p* < 0.001) between the two. The trajectories of LIME weights (Figure 12d) for the three zonal groups of the FMCG treatment show a high degree of robustness: root-volume LIME weights stabilized between −0.42 and −0.44 across all three blocks (SE < 0.015), demonstrating that the dominant negative attribution of root volume in FMCG is not driven by random perturbation of any single block, and constitutes within-sample consistency evidence for the outlier diagnosis in Section 3.5. The LIME three-dimensional weight surface (Figure 12b) further showed that root volume weights formed a maximum inter-treatment difference of 0.85 between FMCG and SACI, SACG, and other treatments, which was much higher than that of other characteristics (generally < 0.15), suggesting that root volume was the most sensitive diagnostic variable separating FMCG from other treatments.

The two classes of methods, SHAP and LIME, are completely independent in their mathematical assumptions (the former is based on a tree-model Shapley value exact algorithm, the latter on a local linear approximation), but give identical diagnostic conclusions about the core feature ordering and the direction of FMCG attribution. This dual validation of independent pathways significantly reduces the risk of bias that may be introduced by a single interpretable method, lends methodological robustness to the identification of milk-ripening root volume as a core predictor in this study, and provides support for the first layer of evidence for the diagnosis of FMCG outlier processing in Section 3.5, which is self-consistent from multiple perspectives within the model.

### 3.5. FMCG Outlier Processing Diagnosis: Four Levels of Evidence Convergence

**Figure 13:**
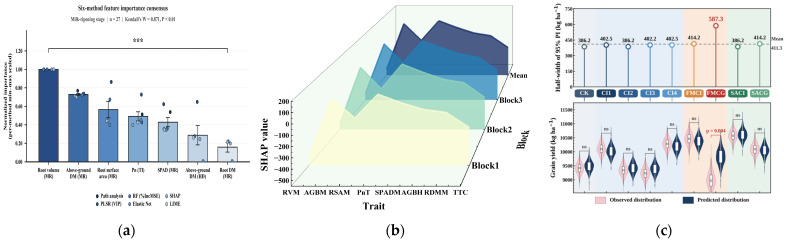
Multi-source evidence convergence for FMCG outlier diagnosis. (**a**) Normalized feature importance from six independent methods (path coefficient, PLSR-VIP, RF %IncMSE, EN |β|, SHAP |mean|, LIME |mean|) for the seven retained predictors at the milk-ripening stage; Kendall’s W = 0.871 (*p* < 0.01). *** *p* < 0.001. (**b**) Three-dimensional SHAP waterfall plot for the FMCG treatment across the three blocks plus block-mean, illustrating the dominant negative contribution of milk-stage root volume (RVM). (**c**) Paired violin plots of observed (pink) and predicted (dark blue) yield distributions for each treatment; “ns” denotes non-significant divergence, whereas FMCG shows significant divergence (paired permutation test on residuals, *p* = 0.003 for RF and *p* = 0.009 for EN). Abbreviations: EN, elastic net; RF, random forest; PLSR, partial least squares regression; VIP, variable importance in projection; %IncMSE, percent increase in mean squared error; SHAP, SHapley Additive exPlanations; LIME, Local Interpretable Model-agnostic Explanations; PI, prediction interval. The convergence of all four evidence layers on FMCG indicates a genuine yield-response outlier—consistent with a hot–anoxic rhizosphere that suppresses root metabolic activity despite intact root morphology—rather than an artifact of small-sample modeling.

To address the outlier nature of the FMCG treatment yield response, this study sequentially diagnosed four levels of intra-model consistency, cross-model consistency, and mechanism-level independent evidence with alignment testing (Figure 13). The four types of independent evidence pathways give conclusions that are fully consistent in direction.

Model internal consistency (Tier 1). The imputation results based on the RF model TreeSHAP algorithm showed that the mean value of root volume contribution during the milk stage period of FMCG was −480 kg ha^−1^, which was the largest negatively contributing feature within FMCG (Figure 13b); and the mean value of root volume weights under the local imputation of LIME was −0.426, and the direction is completely consistent with SHAP (Figure 12d). The LOBO-CV residual triads of RF were +825, +878, and +885 kg ha^−1^ (mean +862.7), respectively. Under RF Jackknife+ conformal prediction, the measured mean values of the three FMCG region groups all fell outside the 95% prediction interval (coverage 0/3; Figure 13c). The EN principal analytical model gives diagnoses in exactly the same direction: −378 kg ha^−1^ for the FMCG milk stage root volume contribution in the standardized coefficient contribution decomposition; +783.4 kg ha^−1^ for the EN LOBO-CV residual mean (three-cohort interval +745 to +812); and −812 for the FMCG milk stage root volume contribution in the standardized coefficient contribution decomposition. 812); The measured mean FMCG value (8976.0 kg ha^−1^) under the EN Jackknife+ conformal prediction falls outside the EN prediction interval [9287, 10,141] (0/3 coverage). Jackknife+, as a conformal prediction framework that does not require data segmentation and has finite-sample coverage guarantees [16], is particularly suitable for detecting systematic deviations in small-sample agronomic data.

Cross-model consistency (layer 2). EN and RF are based on completely different mathematical assumptions (linear + L1/L2 regularization vs. nonparametric integration tree), but give directionally consistent diagnostic results for FMCG: (i) residuals were all systematically positively biased (EN +783.4 vs. RF +862.7 kg ha^−1^, 9.2% difference in magnitude); (ii) Jackknife+ inter-area coverage was 0/3 in all cases, with FMCG being the only treatment among the 9 treatments for which both models reported coverage failures in tandem (Figure 13c); (iii) both SHAP imputation and EN standardized coefficient contribution decomposition identified milky root volume as the largest negative contributing feature (EN −378 vs. RF SHAP −480 kg ha^−1^). This cross-hypothesis consistency is key independent evidence to rule out pseudo-diagnosis due to non-convergence of a single model.

Mechanism Tier Independent Evidence (Tier 3). Rhizosphere microenvironment data (Table 4), based on direct field measurements and not involved in any machine learning modeling, showed that FMCG rhizosphere Eh = −150 mV, TTC reducing power was 38.9% of SACI, and rhizosphere temperature was 2.1 °C higher than CK. The above results are in line with the direction of the mechanistic reports on the decrease in root oxidative activity, the accumulation of reducing ions such as Fe^2+^/Mn^2+^ and the impairment of root uptake function under long-term strongly reducing conditions in the rhizosphere zone of rice [23,28]. The FMCI quasi-experimental control (sharing the same mulch treatment as FMCG but with different water management only) showed a 16.59% (*p* < 0.01) yield increase compared to FMCG, which supports the hypothesis that CI with intermittent desiccation restores the aerobic environment in the rhizosphere zone, and thus mitigates the mechanism of cumulative anaerobic stress induced by mulch warming [25,29].

Distribution-independent permutation test (Level IV). The exact *p*-value based on the RF residuals was 0.003 and the exact *p*-value based on the EN residuals was 0.009 for the 10,000 randomly permuted treatment labels, both well below the 0.05 significance level. Taken together, the four levels of evidence suggest that the yield response pattern of the FMCG treatment did significantly deviate from the overall pattern established by the other eight treatments.

### 3.6. Conformal Forecasting: Quantifying Uncertainty in Treatment-Level Yields

Under the global Jackknife+ conformal prediction (n = 27), the arithmetic mean of the 95% prediction interval (PI) half-widths for the nine treatments was 396.4 kg ha^−1^ for the EN model and 420.2 kg ha^−1^ for the RF model, and the PI half-widths were significantly heterogeneous among treatments (Table S3); The differences in the dispersion of measured and predicted yield distributions among treatments were also clearly seen in the paired violin plot (Figure 13c); the SACI interval was the narrowest (EN half-width 372.8, RF 386.2 kg ha^−1^), and the measured values of the three zones fell into the predicted interval, which indicated that this treatment had the highest yield predictability, consistent with the direction of the highest water use efficiency and the lowest yield coefficient of variation in SACI in Section 3.1. FMCG was the only treatment diagnosed as a coverage failure in both models: the EN model had a lower half-width of 568.4 kg ha^−1^ with 0/3 coverage, while the RF model had a lower half-width of 587.3 kg ha^−1^, also with 0/3 coverage. Empirical coverage across all 27 observations was 88.9% (24/27) for both models, marginally below the 95% nominal level. This bias is consistent with the theoretical expectation of a predictable downward bias in the coverage predicted by the conformation with a small calibration sample. This shortfall is consistent with the method’s guarantee rather than a violation of it: in finite samples Jackknife+ provably ensures a 90% (1 − 2α) lower bound rather than the nominal 95%, so an empirical coverage of 88.9% at n = 27 lies essentially at this theoretical floor, and the companion simulation reproduces it almost exactly (88.4% EN, 87.6% RF), converging to the nominal level by n ≥ 50 (Section 3.7). The treatment-level intervals are accordingly best interpreted as approximately 90–95% intervals at the present sample size.

Under the grouped Mondrian conformal prediction [30], the unaided group (CK + CI1 to CI4, m = 15) had a half-width of 392.4 kg ha^−1^, the mulch group (FMCI + FMCG, m = 6) had a half-width of 521.7 kg ha^−1^, and the improver group (SACI + SACG, m = 6) with a half-width of 401.5 kg ha^−1^. Given that the stratification scale m < 1/(1 − α) = 20 for the mulch and amendment groups, the results of this stratification are only used as an auxiliary diagnostic of residual heterogeneity and do not advocate strict nominal coverage. The degree of deviation from actual coverage due to insufficient stratification size will be quantitatively assessed in Section 3.7 through 1000 replicate simulations.

### 3.7. A Companion Simulation Study: Bias and Variance Behavior of a Diagnostic Framework in Small Samples

Based on the synthetic data of the measured covariance structure and EN standardized coefficient calibration, this study carried out 1000 independent replications under each of the combinations $\left(n\in \{27,50,100\};\alpha_{k}\in \{-400,-800,-1200\}\;\mathrm{kg}\cdot {\mathrm{hm}}^{-2}\right)$ and the results are summarized in Table S4. The simulation design follows the synthetic data validation strategy recommended in small-sample machine learning studies in agronomy, i.e., quantifying the efficacy and bias of the statistical diagnostic framework through Monte Carlo simulations while preserving the covariance structure of the measured data [11,31].

Feature ordering stability. Under the condition of n = 27, the frequency with which root volume at the milk stage was identified as the top feature was 89.4% (EN) and 86.7% (RF SHAP); It rose to 96.2% and 94.8% at n = 50; 99.1% and 98.6% at n = 100. Spearman ρ for the top 3 feature rankings was 0.832 ± 0.118, 0.912 ± 0.071, and 0.964 ± 0.038 for n = 27, 50, and 100, respectively. This result supports the idea that the high degree of consensus (W = 0.871) for the 1st-place feature in the measured n = 27 data is not a sampling fluke, but that there is still reasonable uncertainty in the top 3 internal fine orderings at n = 27.

Analysis of Variance (ANOVA). EN At n = 27 LOBO-CV R^2^ = 0.781 ± 0.082, the training-validation gap is only 0.029 ± 0.011, which is largely plateaued; at n = 100 R^2^ rises only marginally to 0.812 ± 0.031, and the gap drops further to 0.011 ± 0.005RF, at n = 27, yielded LOBO-CV R^2^ = 0.689 ± 0.124 with a train–validation gap of 0.213 ± 0.046; overfitting eased markedly once n ≥ 50, with the gap declining to 0.078 ± 0.021 at n = 100. This pattern of behavior quantitatively validates the appropriateness of this study’s methodological choice to use EN as the primary analytical model and RF as a complementary nonlinear control. EN exhibits lower variance and a smaller training-validation gap under small-sample conditions, which is consistent with the theoretical expectation that regularized linear models achieve a better bias-variance trade-off compared to nonparametric ensemble methods in high-dimensional, low-sample-size (high-p-low-n) scenarios [32].

Jackknife+ practical experience coverage. Coverage gradually converges to the nominal level as n increases: 88.4% ± 4.2% for EN and 87.6% ± 5.1% for RF at n = 27 (about 6 to 7 percentage points below the nominal level); 92.1% ± 3.1% and 91.4% ± 3.6%, respectively, at n = 50; and 94.2% ± 2.4% and 93.8% ± 2.7%, respectively, at n = 100. The empirical coverage of the measured EN and RF at n = 27 (both 88.9%) is in high agreement with the simulated mean values, indicating that the low measured coverage mainly stems from the inherent limitations of the small samples rather than a modeling problem. Barber et al. demonstrated theoretically that Jackknife+ guarantees a 1 − 2α coverage lower bound (i.e., the empirical coverage may be lower than 1 − α when n is finite), and the simulation results of the present study are quantitatively consistent with this theoretical expectation [16].

Ranking test efficacy and Mondrian coverage. The efficacy of the alignment test for detecting FMCG-like outlier treatments increased monotonically with the absolute value of α_k_ versus n. At n = 27, the corresponding efficacies for α_k_ = −400, −800, and −1200 kg ha^−1^ are 0.41/0.39, 0.78/0.76, and 0.94/0.93 (EN/RF); the measured FMCG residual of about −860 kg ha^−1^ falls in the α_k_ = −800 region; the efficacy of α_k_ = −800 at n = 50 rises to 0.92/0.91. The actual coverage rates of Mondrian cluster-based conformal prediction at different clustering levels were: 76.4% ± 11.8% for m = 3, 84.7% ± 8.2% for m = 6, and 91.2% ± 5.4% for m = 15. These results quantitatively confirm that the actual coverage of the film group and the modifier group (m = 6) is approximately 85%. The grouping results should only be used as an auxiliary diagnostic tool for residual heterogeneity and should not be interpreted statistically in terms of strict nominal coverage.

Reliability of cross-model consistency diagnostics. Under the conditions α_k_ = −800 and n = 27, the frequency with which EN and RF provided consistent diagnostic results for the FMCG category was 81.3%; under the condition α_k_ = −1200, this figure rose to 94.7%. These results indicate that, at the observed FMCG residual range (α_k_ ≈ −860 kg ha^−1^), the reliability of cross-model consistent diagnosis is approximately 80% to 85%. This reliability, reinforced by independent evidence at the mechanism level in Section 3.5 (three actual measurements of rhizosphere Eh, TTC reducing power, and rhizosphere temperature), achieves the strength of evidence needed to establish outlier treatment identification in a small-sample field trial. This multilayered evidence convergence strategy, which combines statistical model diagnostics with independent mechanism evidence, follows the methodological principle of “model interpretation must be premised on domain knowledge validation”, which has been advocated in the recent interpretable machine learning literature [11,33].

## 4. Discussion

### 4.1. Milk-Ripening Root Vigor as a Key Predictor of Yield Formation in Cold Water-Saving Rice

Six independent modeling approaches converged on milk-stage root volume as the top yield predictor (Kendall’s W = 0.871, *p* < 0.01), a ranking confirmed as ~89% stable by 1000 Monte Carlo replicates.

Importantly, this multi-method consensus establishes root volume as a robust statistical predictor whose ranking is independent of specific model assumptions, but does not in itself constitute proof of physiological causation. The following evidence—path-analytic mediation (Section 3.3), independent rhizosphere measurements deliberately excluded from the ML feature set (Section 3.2), and the FMCI–FMCG quasi-experimental comparison (Section 3.5)—collectively elevates this association from simple correlation to a mechanistically plausible predictive relationship.

The physiological plausibility of this finding can be elucidated by the special phenological–thermal constraints of cold-region japonica rice. The effective cumulative temperature of rice crops in the Sanjiang Plain is only about 2300 °C-d, and the grouting window is significantly compressed by low-temperature stress, so that the root function during the grouting period has a higher weight in the yield formation chain than in warmer rice areas. A two-year temperature control experiment conducted by Guo et al. [4] in 2023 and 2024 on ‘Longjing 31’ confirmed that low-temperature stress significantly suppressed the net photosynthetic rate at the tillering, panicle and heading stages, with the greatest reduction at the panicle stage. Sun et al. [34] further showed by ^13^C and ^15^N double labeling that low-temperature stress at the heading stage resulted in a significant decrease in the proportion of carbon and nitrogen assimilates partitioned to the grain, and a rise in stem and leaf retention, which strengthened the regulatory weight of root signaling on source–sink–transport coupling. The above evidence supports the rationality of this study to identify root function as a key node for yield formation in cold-region japonica rice.

The molecular mechanisms by which root function regulates seed filling during the filling period have been revealed by several recent studies. Cao et al. [35] found that the expression of sucrose synthase (SUS) and starch synthesis-related genes was significantly suppressed under low temperature stress during the filling stage, and that the root hormone–carbohydrate coupling pathway constituted the core mechanism for the differences in cold tolerance in cold-region japonica rice based on a comparison of the transcriptomes of eight chilling-sensitive and chilling-tolerant varieties. Guo et al. [36] further pointed out, based on a three-year field experiment from 2017 to 2019, that for every 10 °C-d increase in cumulative coolness days during the grouting period, rice yield decreased by 1.7% to 2.2%, and that the mechanism of the effect on yield and quality was different between early grouting and late grouting. The flux analysis in this study showed that the indirect flux coefficient of aboveground DM transferred to yield via root volume amounted to 0.182, accounting for 37.8% of its total effect, which was consistent with the indication that the regulatory weight of root function during the filling period was significantly higher here than that in the warmer rice region under the source–sink–transport framework described above. It should be noted that inter-feature correlations among root traits and aboveground biomass (r = 0.47, *p* < 0.05; Figure 9c) raise a legitimate concern regarding multicollinearity-inflated feature importance. However, the dominance of root volume survived three independent safeguards against multicollinearity. First, the partial correlation between root volume and yield remained significant after controlling for all other retained features (r_partial = 0.642, *p* < 0.01), confirming that the association is not driven by shared variance. Second, the six ranking methods differ fundamentally in their sensitivity to correlated predictors: EN imposes L1/L2 penalties, PLSR projects onto orthogonal latent variables, and path analysis screens by VIF, yet all converged on root volume as the top-ranked predictor (Kendall’s W = 0.871). Third, the most collinear traits, specifically root surface area and root DM, were excluded by VIF screening or shrunk to zero by EN regularization (Table 5), while root volume was retained by every method. Taken together, these results indicate that the top ranking of root volume reflects a genuine predictive signal rather than an artifact of multicollinearity.

A two-dimensional partial dependence analysis using random forests revealed that when root volume at the milk stage was coupled with high values of aboveground dry matter (DM), the yield gain (approximately 2700 kg ha^−1^) exceeded the independent sum (approximately 1930 kg ha^−1^) by about 770 kg ha^−1^, indicating a statistically significant superadditive relationship. This nonlinear superadditive association is a statistical description conditional on the RF model structure and the observed data in this study, and does not, in itself, constitute rigorous proof of physiological causation. However, the convergence of this statistical pattern with the independent rhizosphere Eh gradient (SACI +80 mV vs. FMCG −150 mV), the TTC reductive activity contrast (SACI 432 vs. FMCG 168 μg g^−1^ h^−1^), and the quasi-experimental FMCI–FMCG yield divergence collectively elevates the finding to the level of a mechanistically plausible association. Final causal confirmation will require ^13^C pulse-chase labeling coupled with source–sink manipulation experiments in future work. However, the synergistic enhancement of root volume up to 80.81 cm^3^- hill^−1^ (121.5% higher than CK) and aboveground DM 32.84 g hill^−1^ (23.7% higher than CK) at the milk stage of the SACI treatments fell exactly in this nonlinear high gain interval. Xiao et al. [21], in a two-year field experiment (2023–2024), reported that AWD elevated grain yield, WUE, and economic efficiency by 12.07%, 12.27%, and 29.18%, respectively, through enhanced photosynthate production and redistribution and improved root traits (deep-root distribution, root oxidative activity, and proportion of aerenchyma). This is in close agreement with the “coupled high-biomass × high-root-function” advantage observed during the grain-filling stage in the present study. 

Excessive ineffective tillering in the CI3 treatment (60% θfc CI) provided direct field evidence of a negative mechanism of excessive water deficit. It had 158 more peak tillers-m^−2^ than CI4, but only 57.3% effective panicle retention. Dou et al. [37] reached a directionally consistent conclusion in a two-year field experiment with ‘Longjing 31’ in the black-soil rice region of the Sanjiang Plain: grain yield declined by 14.9–17.3% when the soil water-potential threshold deepened from −10 kPa to −30 kPa. Combined with the present finding that the filled-grain percentage of CI3 (84.6%) was significantly lower than that of CI4 (86.9%), these results jointly indicate a physiologically defined lower safety threshold for CI in cold rice systems.

### 4.2. Diagnosis of FMCG Outlier Processing: From Morphological Integrity to Functional Collapse of Metabolic Inactivation

FMCG exhibited moderate instantaneous Pn at the milk stage (14.90 μmol CO_2_ m^−2^ s^−1^, above CK and CI3) yet the lowest grain yield among all nine treatments (8976.0 kg ha^−1^). This dissociation indicates that single-time-point Pn does not translate directly into season-long assimilate accumulation. Despite intact photosynthetic capacity and root morphology, FMCG yield collapsed through functional metabolic inactivation of the root system. This functional decline is also reflected in the rate of decline at the source end: the SPAD retention rate during the milk ripening stage of FMCG (78.4%) was approximately 10 percentage points lower than that of SACI (87.0%). This corresponds chronologically with the decline in TTC recovery capacity to 38.9% of that of SACI, suggesting that the synergistic destabilization of “decreased continuous supply capacity at the source + decreased root translocation capacity” was the functional mechanism underlying the yield collapse.

Field measurements of the rhizosphere microenvironment provide direct evidence for this mechanism. The rhizosphere Eh value of FMCG is −150 mV, which corresponds to strongly reducing conditions and is well below the Eh threshold required for normal rice root growth. We note that dissolved O_2_ was not measured directly; rhizosphere oxygen status was inferred from in situ Eh, supported by root TTC-reducing activity. In submerged paddy soils Eh is the standard electrochemical indicator of aeration status, as O_2_ is the first species removed along the thermodynamic reduction sequence (O_2_ → NO_3_^−^ → Mn^4+^ → Fe^3+^ → SO_4_^2−^); the FMCG value of −150 mV thus lies well within the strongly reducing range in which molecular O_2_ is no longer thermodynamically stable, while the collapse of TTC activity to 38.9% of SACI independently confirms this oxygen-limited state. We acknowledge that Eh reflects the bulk rhizosphere rather than the root surface, where radial oxygen loss may sustain micro-oxic zones; ‘anoxic’ therefore denotes an O_2_-depleted bulk rhizosphere, and direct O_2_ profiling (planar optodes or O_2_ microelectrodes) would be needed to resolve the fine-scale gradient at the root surface. Pedersen et al. [38] systematically summarized that rice maintains a rhizosphere local oxidative environment through aeration tissue formation with radial oxygen loss (ROL); Maisch et al. [39] further confirmed by in situ optical measurements that the spatiotemporal pattern of ROL directly drives rhizosphere Fe redox processes with Fe^3+^ patch formation. Prolonged exposure of the rhizosphere to strongly reducing conditions will lead to a decrease in root oxidative activity, accumulation of reducing ions such as Fe^2+^ and Mn^2+^ and direct impairment of root uptake. Xiao et al. [21] reported that AWD increased root oxidative activity, the proportion of deep root distribution and the proportion of aerated tissues simultaneously by elevating the rhizosphere dissolved oxygen concentration and Eh as compared to conventional flooding irrigation, thereby increasing yield and WUE by 12.07% and 12.27%, respectively. This mechanistic pathway is fully consistent with the direction of the extreme 230 mV difference between SACI (Eh = +80 mV) and FMCG (Eh = −150 mV) observed in this study.

The combination of FMCG treatment with plastic mulch for temperature elevation (resulting in a root zone temperature 2.1 °C higher than that of the control) and prolonged waterlogging created a high-temperature, anaerobic root zone microenvironment, and this anoxic stagnation is further aggravated on albic soil, whose low-permeability argic confining layer impedes vertical drainage and prolongs subsurface waterlogging under continuous flooding. Consequently, although the root system retained its structural integrity, its metabolic activity severely declined, as evidenced by a TTC reduction capacity that was only 38.9% of that of SACI. The phenomenon that the yield response of film mulching was in the opposite direction under different water management practices is highly consistent with recent reports from film mulching coupling studies in cold rice areas. Huang et al. [7], based on a meta-analysis of 13 crops across the country, pointed out that improvements in yield, water use efficiency and nitrogen fertilizer bias productivity of ground film cover in low temperature, semi-arid and arid zones were significantly greater than those in warm and humid areas. Zhao et al. [40] observed that biodegradable film in combination with non-flooded cultivation significantly enhanced water productivity (30% in 2021 and 52.85% to 60% in 2022) in a two-year direct-seeded rice trial using the ‘Baonong 5’ variety in a cold rice cropping area in northern China (Zalaid Banner, Inner Mongolia, 46°41′ N), which is in the same direction as the high-yield performance of the FMCI in this study. Multiple independent lines of evidence systematically point to the same conclusion: the yield-enhancing benefits of mulching in cold rice crops are highly dependent on synergistic configurations of water management practices, and the application of mulching in isolation without adjustments to water management may be not only unhelpful, but also detrimental.

The results of the FMCI quasi-experimental control mulch with CI, yielding 16.59% (*p* < 0.01) more than FMCG, provided key supporting evidence for mechanism inference. The two shared mulch treatments but differed only in water management practices, and the periodic aerobic and mild water deficit rhizosphere environment created by CI intermittent drop-drying contrasted with the cumulative stagnant anoxic environment created by continuous flooding of FMCG. This control is a quasi-experimental design rather than a proof of causality in the sense of a strictly randomized, manipulated trial, but the multi-pathway directional consistency with the available mechanistic evidence significantly reduces the likelihood of alternative explanations.

The four-layer evidence convergence framework used in this study provides varying strengths of support for the mechanism identification. The first level involves internal consistency across multiple perspectives within the same model, which is a necessary condition; the second level involves cross-model consistency between EN and RF (residuals of +783.4 vs. +862.7 kg ha^−1^, with consistent directions), ruling out the risk of a false diagnosis due to the RF model failing to converge; the third level involves independent measurements at the mechanistic level and quasi-experimental controls using the FMCI, providing an independent evidence pathway outside the model; and the fourth level consists of distribution-independent permutation tests (*p* = 0.009 for the EN-based model and *p* = 0.003 for the RF-based model). The simulation study showed an alignment test efficacy of approximately 0.78 and a cross-model agreement diagnostic reliability of approximately 81% at the measured FMCG residual magnitude (α_k_ ≈ −860 kg ha^−1^). Both types of statistical evidence had a nonzero false-positive likelihood, and the robustness of the final diagnostic conclusions was largely consolidated by mechanism-level independent evidence. We emphasize, however, that the present rhizosphere measurements (Eh, TTC reductive activity, and temperature) and the FMCI quasi-experimental contrast constitute strong corroborative rather than direct causal evidence for “hot-anoxic rhizospheric inhibition.” Definitive confirmation will require targeted follow-up experiments, including direct quantification of rhizosphere Fe^2+^/Mn^2+^ and reduced phytotoxins along the redox gradient, in situ measurement of dissolved oxygen and radial oxygen loss, anatomical quantification of aerenchyma and the radial-oxygen-loss barrier, and a controlled factorial design that orthogonally varies root-zone temperature and oxygen status to separate their respective contributions.

From a production perspective, the findings of this study suggest that, under similar site conditions on the Sanjiang Plain, plastic mulch should be used in conjunction with CI and intermittent drying; the combination of plastic mulch and continuous flooding should be explicitly avoided. For production monitoring, root-zone Eh and root TTC-reducing power should be incorporated into the routine diagnostic index system for the milk stage.

### 4.3. Physio-Ecological Mechanisms of SACI Synergistic Dominance and Their Agronomic Significance

The SACI treatment achieved a three-dimensional synergistic advantage of yield, water saving and WUE: 12.3% yield increase (*p* < 0.05), 17.0% water saving and 35.3% WUE enhancement over conventional flood irrigation. The lift observed in this trial is at the upper end of the range reported in the combined evidence of recent years. Gao et al. [5] reported in a global meta-analysis based on 437 papers that AWD has a significant advantage in water saving and GHG emission reduction, but the yield is slightly reduced, and AWD with appropriate thresholds can realize a win–win–win situation of “yield–water saving–emission reduction”. Xiao et al. [21] reported that AWD increased yield and WUE by 12.07% and 12.27%, respectively, in a two-year field trial from 2023 to 2024. The SACI in this study was comparable to that reported by Xiao et al. [21] in terms of yield and WUE improvement, but the water saving was slightly lower. This difference may reflect the site characteristics of lower evapotranspiration in the Sanjiang Plain relative to tropical rice areas, and the marginal benefits of CI and water saving are limited by shorter fertility and lower evapotranspiration driving force. It is worth noting that the 2.96% marginal increase in yield observed with SACI compared to CI4 (75% θfc CI alone) (*p* < 0.05) indicates that soil conditioners can still yield quantifiable synergistic benefits when used in conjunction with water-saving CI.

The synergistic benefits of SACI may be attributable to complementary functions of the composite amendment’s individual components—humic acid, diatomaceous earth, bentonite, and trace elements—although the following mechanistic pathways are inferred from product composition and published literature rather than directly measured in this experiment. First, the carboxyl and phenolic hydroxyl groups of humic acid are known to chelate reducing ions such as Fe^2+^ and Mn^2+^, which could help alleviate rhizosphere redox imbalance. Faria de Souza et al. [41] demonstrated that humic acid can synergistically promote rice root development through a ROS-dependent pathway in combination with hormone signaling via ROS-scavenger and hormone signaling pathway inhibitor experiments. Rice root development increased root dry weight by 27% and lateral root length by 20%. Hartina et al. [42] further demonstrated that humic-acid application alone significantly increased cation exchange capacity, total organic carbon, phosphorus utilization efficiency (+50%), and phosphorus agronomic efficiency (153 kg kg^−1^) in acidic paddy soils, providing direct evidence for humic-acid-mediated improvement of soil nutrient dynamics in rice systems. Second, the porous structure of diatomaceous earth may provide a buffer space for both water and gas phases while potentially releasing soluble silicon available for rice uptake; rice is a typical silicon-loving crop. Fu et al. [43] confirmed in field trials at two sites in Hainan that the synergistic application of Zn and Si can simultaneously improve rice yield and lodging resistance. Jiang et al. [44] further demonstrated in a drought-stressed cultivation system that an appropriate amount of silicon fertilizer (SiO_2_ 45 kg·ha^−1^) can synergistically optimize both yield and stem lodging resistance, which holds particular value for rice cultivation in cold regions. Third, the interlayer water-holding capacity of bentonite may slow the rate of sudden moisture decline in the rhizosphere, potentially buffering root function during the controlled irrigation drying phase. Finally, the supplementation of trace elements such as Fe, Zn, and Mn may partially alleviate trace element deficiencies commonly found in cold-region podzolic soils. These four putative pathways are expected to be most effective under the periodic aerobic and mildly water-deficient rhizosphere conditions created by controlled irrigation at 75% θfc, though their individual contributions remain to be quantified.

It has also been shown that the coupled synergistic effects of water-saving irrigation and different types of amendments have cross-field commonalities. Zhang et al. [6] reported in the Northeast China black soil rice-growing region that a combination of biochar and CI can simultaneously improve root morphological parameters and nitrogen use efficiency. Specifically, the CB2 treatment (12.5 t·hm^−2^ biochar combined with CI) resulted in increases of 16.45%, 39.42%, 24.48%, and 16.45% in yield, NUE, NAE, and NPFP, respectively, compared to conventional flood irrigation. This study extended this synergistic mechanism to composite soil conditioners containing humic acid, diatomaceous earth, and bentonite, further demonstrating that different types of soil conditioners can all form synergistic effects with water-saving CI through common pathways. An important limitation must be acknowledged: this study did not directly measure soil aggregate structure, bulk density, porosity, microbial community composition, or plant nutrient uptake dynamics. The four mechanistic pathways discussed above are therefore working hypotheses inferred from the known composition of the composite amendment and the existing literature, not empirically confirmed causal mechanisms. Verification through CT-scanning-based soil microstructure analysis, 16S rRNA and metagenomic sequencing, enzyme activity profiling, and plant tissue nutrient analysis under the same treatment combinations is a priority for future work.

It is worth pointing out that the coverage of the available AWD Meta-analyses is dominated by tropical and subtropical rice regions, and the systematic evidence on the coupling of CI thresholds and amendments in cold rice regions is relatively weak [5,21]. Given the central role of the Northeast rice-growing region’s cultivated area and production capacity in the national rice industry, as well as the crisis in groundwater sustainability caused by the expansion of rice cultivation in this region [45], the safe CI threshold (75% θfc) and the strategy for combining soil conditioners under similar meadow podzol soil conditions on the Sanjiang Plain, as proposed in this study, provide direct production guidance for the sustainable development of rice cultivation in the region. From a production-oriented perspective, the SACI strategy translates into the following actionable recommendation for cold-region rice systems on albic soils of the Sanjiang Plain: (i) Maintain the CI lower threshold at ≥ 75% θfc. (ii) Apply a humic-acid-based composite amendment (humic acid ≥ 30% + diatomite ≥ 15% + bentonite ≥ 20%, supplemented with Fe/Zn/Mn) at 3.0 t ha^−1^ as a basal application incorporated into the 0–15 cm layer 7 d before transplanting. (iii) For the cultivar ‘Longjing 31’ transplanted at 23.8 × 10^4^ hills ha^−1^ with three seedlings per hill, the “film mulching × continuous flooding” combination should be explicitly avoided. The aforementioned multidimensional chain of evidence linking root morphology, rhizosphere oxidative status, and yield enhancement provides mechanistic support for Hypothesis H1 of this study.

### 4.4. Methodological Assessment Under the Companion Simulation Study

This study employs a dual-verification paradigm combining simulation studies and real-world data to actively quantify the bias-variance behavior, actual coverage of conformal prediction, the power of permutation tests, and cross-model consistency of the diagnostic framework used, thereby making the methodological applicability boundaries themselves a key contribution of the paper.

The simulation study first validated the rationality of the methodological decisions at the model selection level: the quantitative diagnosis that EN had essentially converged at n = 27 while RF had not yet fully converged supported the choice of using EN as the primary analysis and RF as a nonlinear complementary control, thereby avoiding the risk of overfitting associated with using a non-converged model as the primary basis for inference. Based on this, the high level of consensus in feature ranking (W = 0.871) was confirmed not to be a sampling fluke—the stability of the top-ranked feature was approximately 89%, but there remained reasonable uncertainty regarding the precise order of the top three features. This finding is consistent with the recommendations proposed by Bouni et al. [27] that explainable machine learning in agricultural contexts should combine multi-model consensus with robustness diagnostics. The SHAP representativeness estimation framework under nested cross-validation proposed by Scheda and Diciotti et al. [46] also demonstrates superior ranking stability compared to point estimates across multiple resampling runs.

At the level of uncertainty quantification, the actual coverage under the Jackknife+ covariate forecast is about 6 to 7 percentage points lower than the nominal 95%, a behavioral pattern that suggests that the covariate forecast should be treated as an approximate 95% rather than a strict nominal confidence interval. This conclusion is fully consistent with the findings reported by Kakhani et al. [17] in their continental-scale remote sensing inversion of soil organic carbon in Europe—specifically, that the conformal prediction coverage exhibits predictable deviations as the calibration sample size decreases. The theoretical framework of Jackknife+ was established by Candes et al. [16], and its coverage is guaranteed to be 1 − 2α rather than 1 − α under small sample size conditions. Furthermore, the rank-sum test yielded a power of approximately 0.78 for detecting outliers in FMCG magnitude (residual ≈ −860 kg ha^−1^), with cross-model diagnostic reliability of approximately 81%—neither type of statistical evidence can independently support a conclusion of causality; they must be used in conjunction with independent evidence at the mechanistic level. This is precisely the methodological basis for the four-tier evidence convergence framework developed in this study.

Compared to the analytical framework of traditional one-way ANOVA and descriptions of physiological mechanisms, the added value of the methodological combination used in this study can be summarized in four dimensions: Nested cross-validation and multi-method consensus ensure that the identification of key predictors is independent of specific model selection or data partitioning. The convergence of multiple sources of evidence—including cross-model residuals, conformal prediction coverage, permutation tests, and evidence from the mechanism layer—elevates outlier detection from qualitative observation to reproducible statistical inference; The difference in the half-width of conformal predictions provides a quantitative basis for assessing the robustness of yield forecasts in production decision-making; simulation studies actively quantify the actual performance of the method, thereby making its methodological limitations transparent.

### 4.5. Areas for Further Development and Outlook

This study has yielded a series of findings regarding the regulatory mechanisms of root function in cold-region water-saving rice cultivation and the development of a methodological diagnostic framework. Based on the conclusions of this study, the following three areas warrant further in-depth exploration.

First, regarding the detailed mechanistic analysis of the action pathways of soil amendments, this study primarily elucidated the synergistic mechanisms between the composite amendment of humic acid, diatomaceous earth, and bentonite and water-saving CI by inferring from product components and interpreting field phenotypes; however, intermediate process indicators such as changes in soil aggregate structure, bulk density, and porosity, as well as microbial community structure and the abundance of functional genes, have not yet been directly measured. Future work could combine CT-scanning-based soil microstructure analysis, 16S rRNA and metagenomic sequencing, and enzyme activity profiling to systematically characterize the physicochemical and biological pathways through which soil conditioners reshape the rhizosphere microenvironment under different water management regimes, thereby providing a mechanistic basis for the precise optimization of soil conditioner formulations.

Second, regarding the isotopic validation of causal mechanisms, this study used pathway analysis and two-dimensional RF PDP to reveal the epistatic contribution of root volume at the milk stage and aboveground DM to yield; however, the dynamics of assimilate allocation between sources and sinks during the grain-filling stage still require direct tracing. A follow-up source-reservoir manipulation experiment of ^13^C and ^15^N dual labeling combined with source leaf clipping and reservoir organ shading can be designed to quantitatively resolve the transfer efficiency and spatial–temporal partitioning pattern of carbon and nitrogen assimilation products to seeds during the filling period under SACI treatments, and to further advance the statistical correlations of the present study into a chain of evidence for physiological causation.

Third, in terms of iterative refinement of the methodological framework, the five-layer framework of nested cross-validation, multi-method consensus, conformal prediction, and permutation testing with mechanism-level independent evidence established in this study has demonstrated diagnostic robustness under field trial data conditions. In future work, we can further evaluate the conditional coverage performance of Mondrian’s grouped conformal prediction on a larger sample size and explore extending this framework to root-yield coupling diagnostics and outlier identification for other crops, thereby promoting the standardized application of interpretable machine learning methods in the field of agronomy. In addition, due to the inherent limitations of the field trial design with multiple treatments, the effective sample size for machine learning modeling was n = 27 (combined average over two years). Although the bias-variance behavior of the methodological diagnosis, the conformal prediction coverage, and the power of the permutation test for this sample size have been actively quantified based on 1000 paired Monte Carlo simulations (Section 3.7), the uncertainty in the fine-grained ranking of the top three features (Spearman’s ρ = 0.832 ± 0.118) and the impact of sample size limitations on the coverage of conformal prediction conditions in the Mondrian grouping still constitute the objective lower bounds of this study’s methodological scope. Subsequent sample sizes of n ≥ 50 in multiyear multisite extension trials could further tighten the uncertainty boundaries of the above diagnostic metrics.

## 5. Conclusions

This study conducted a two-year field trial from 2024 to 2025 at the Qinglongshan National Agricultural Science and Technology Park on the Sanjiang Plain in Heilongjiang Province. Addressing the dual bottlenecks of water scarcity and low-temperature heat limitation faced by cold-region japonica rice, the study systematically evaluated the synergistic effects of nine “irrigation method × supplementary measure” combinations on yield, water use efficiency (WUE), and root physiological traits. An interpretable machine learning diagnostic framework was developed, featuring elastic net as the primary analysis method, random forest as a nonlinear control, and supplemented by conformal prediction, permutation testing, and supporting simulation studies. The main findings are summarized below around the three research objectives of quantifying coupled treatment effects and identifying optimal solutions, identifying core physiological predictors, and diagnosing yield response outlier treatments.

First, the combination of soil conditioners and 75% θfc-CI (SACI) achieved a three-dimensional synergy of yield, water savings, and WUE. Compared with conventional flood irrigation (CK), SACI increased yield by 12.3%, reduced water use by 17.0%, and improved WUE by 35.3% (all *p* < 0.05), and exhibited the highest interannual yield stability. Tukey HSD tests confirmed that SACI yield was significantly higher than all treatments except FMCI (*p* > 0.05); SACI is recommended as the preferred strategy because it simultaneously achieved the highest yield, WUE, and yield predictability. Comparison of the gradient of the lower limit of CI showed that 75% θfc was a safe threshold for balancing yield stability and water saving benefits; excessive water deficit (60% θfc) would stimulate ineffective tillering, reduce effective panicle retention and fruit set, and should not be used as a recommended threshold for production. The above results support the research hypothesis H1.

Second, milk-stage root volume was identified as the most robust statistical predictor of grain yield in cold-region japonica rice, supported by convergent evidence from independent rhizosphere measurements. The six independent interpretable modeling methods consistently ranked this trait first among the seven retained predictors, reaching a high level of concordance (Kendall’s W = 0.871, *p* < 0.01), and the companion Monte Carlo simulation confirmed that this top ranking was retained in approximately 89% of replicates at n = 27. This finding elevates root volume from a classical physiological descriptor to a quantifiable, reproducible, and field-diagnosable predictor of yield—one whose causal contribution is mechanistically plausible but remains to be confirmed by direct isotopic tracing—thereby providing a high signal-to-noise monitoring target for the precision management of water-saving rice in cold regions. Root TTC reducing activity at the milk stage was strongly correlated with grain yield (r = 0.79, *p* < 0.001); the assay is standardized and can be completed in ~4 h in the field, rendering it directly applicable for production-scale diagnostic monitoring.

Third, the film-mulching × continuous-flooding treatment (FMCG) was diagnosed as a yield-response outlier, the underlying mechanism being functional inactivation of root metabolism under a hot–anoxic rhizospheric microenvironment, rather than defects in root morphology. Diagnosis of cross-model residual consistency, field measurements of the rhizosphere microenvironment (Eh = −150 mV, TTC reducing power reduced to 38.9% of SACI, and rhizosphere temperature 2.1 °C higher than CK), and non-distribution-dependent permutation tests all point in the same direction across four levels of evidence, indicating that FMCG deviates from the general pattern; The yield increase of 16.59% (*p* < 0.01) observed in the “mulch film combined with CI” quasi-experimental control (FMCI) treatment compared to the FMCG treatment further supports this proposed mechanism. The above results confirm research hypothesis H2 and provide direct field evidence that “the warming effect of plastic mulch becomes a yield-limiting factor when there is a lack of synergy in water management.”

Fourth, regarding the limitations of this study, the companion simulation study quantitatively assessed the methodological boundaries of the proposed diagnostic framework, making the range of applicability itself one of the outputs of this study. The 1000 Monte Carlo replicates clarified that the framework is applicable under the following preconditions: (a) a balanced, multi-treatment factorial design with clear controls (e.g., RCBD with ≥3 replicates); (b) a sample size of n ≥ 27 treatment-level means, with between-treatment CV substantially exceeding within-treatment CV; (c) availability of independent mechanism-level evidence (e.g., rhizosphere measurements not used in ML modeling) to corroborate statistical outlier diagnoses; and (d) access to specialized statistical expertise for implementing nested cross-validation, conformal prediction, and permutation testing. The simulations further indicated that Mondrian group-based conformal prediction should only be used to diagnose residual heterogeneity when the sample size per stratum, m, is less than 20, and that permutation tests and cross-model consistency diagnostics alone are insufficient—they must be reinforced by independent mechanistic evidence to support robust outlier identification. This evaluation paradigm focuses on the bias-variance behavior of the diagnostic tool itself, providing a reusable template for quantifying reliability in similar studies.

Based on the above conclusions, this study recommends the use of SACI as the preferred strategy for water conservation and yield enhancement in similar meadow podzolic soils on the Sanjiang Plain. The lower limit for CI must be strictly maintained at no less than 75% of θfc; plastic mulch technology must be used in conjunction with intermittent drying during CI, and the combination of “plastic mulch with continuous flooding” should be explicitly avoided; For production monitoring, rhizosphere Eh (measured using the platinum electrode method, which takes approximately 30 min in the field) and root TTC reducing power at the milk stage should be incorporated into the routine diagnostic indicator system. Given the central role of the Northeast rice-growing region’s cultivated area and production capacity in the national rice industry, as well as the challenges to groundwater sustainability posed by the expansion of rice cultivation in the region, the optimal scenario identified in this study provides direct guidance for the sustainable development of rice cultivation in the region.

At the methodological level, the five-component diagnostic paradigm established in this study—multi-method consensus under nested cross-validation, conformal prediction, permutation testing, independent mechanistic evidence, and companion simulations—provides a reusable methodological template specifically suited to multi-treatment, small-sample field trials (n ≈ 20–60) with balanced factorial structure and available domain-knowledge validation. Its application to observational datasets, unbalanced designs, or scenarios lacking independent mechanistic evidence would require additional methodological adaptation and should not be assumed without further validation.

Future work will be deepened along three main avenues: detailed mechanistic analysis of how composite soil amendments reshape the rhizosphere microenvironment; isotopic causal validation of source–sink dynamics during the grain-filling stage; and the cross-scenario application of the methodological framework.

## Figures and Tables

**Table 1:** Nine coupled “irrigation regime × auxiliary practice” treatments applied in 2024 and 2025.

Treatment	Irrigation Regime	Auxiliary Practice	CI Lower Limit (% θfc)	Water Layer Limit (mm)	Design Rationale
CK	Continuous flooding (CF)	None	—	80–100	Conventional local control
CI1	Controlled irrigation (CI)	None	80	30–50	Mild water regulation
CI2	Controlled irrigation (CI)	None	70	30–50	Moderate water regulation
CI3	Controlled irrigation (CI)	None	60	30–50	Severe water regulation (exploring threshold floor)
CI4	Controlled irrigation (CI)	None	75	30–50	Mild–moderate regulation (recommended threshold)
FMCI	Controlled irrigation (CI)	Film mulching (FM)	75	30–50	FM × CI coupling
FMCG	Continuous flooding (CF)	Film mulching (FM)	—	80–100	FM × CF coupling
SACI	Controlled irrigation (CI)	Soil amendment (SA)	75	30–50	SA × CI coupling
SACG	Continuous flooding (CF)	Soil amendment (SA)	—	80–100	SA × CF coupling

θfc = field capacity (volumetric water content, 38.2% *v*/*v*). CK maintained an 80–100 mm standing water layer from peak tillering to the wax-ripening stage (20–40 mm shallow layer from transplanting to early tillering). CI treatments were re-irrigated to a 30–50 mm layer once the 0–20 cm volumetric water content dropped to the designated lower threshold, then allowed to dry down naturally. CK and all CG-suffixed treatments were drained 10 d before physiological maturity; CI treatments stopped irrigation 7 d before physiological maturity. FM and SA were crossed only with the 75% θfc CI level (the optimal threshold identified from the CI1–CI4 gradient) or with CF, and were not combined with the 60%, 70%, or 80% θfc CI levels.

**Table 2:** Overview of measurement parameters, target growth stages, and analytical methods used to characterize canopy physiology, root morphology, rhizosphere microenvironment, and yield-related variables of rice.

Measurement Category	Parameter	Growth Stage/Sampling Time	Instrument/Method
Canopy physiology	Net photosynthetic rate (Pn)	Peak tillering, jointing–booting, heading–flowering, and milk stages	LI-6800 portable photosynthesis system (LI-COR Biosciences, Lincoln, NE, USA)
Canopy physiology	SPAD value	Same four stages as above	SPAD-502Plus chlorophyll meter (Konica Minolta, Inc., Tokyo, Japan)
Canopy physiology	Tiller dynamics/dry matter accumulation	Every 5 d; each growth stage	Fixed quadrat sampling; oven-drying at 80 °C to constant weight
Root morphology	Total root length, root surface area, root volume, mean root diameter	Milk stage	Epson 12000XL scanner (Seiko Epson Corp., Suwa, Japan) with WinRHIZO Pro (version 2019a, Regent Instruments Inc., Quebec City, QC, Canada)
Rhizosphere microenvironment	Rhizosphere redox potential (Eh)	Milk stage (in situ)	FJA-6 ORP meter (Shanghai INESA Scientific Instrument Co., Ltd., Shanghai, China) with Pt/SCEs
Rhizosphere microenvironment	Root TTC reduction activity	Milk stage	TTC method (37 °C, 3 h, 485 nm)
Rhizosphere microenvironment	Rhizosphere temperature	Grain-filling stage (5–15 August)	EM50 data logger with Echo-T probes (METER Group, Inc., Pullman, WA, USA)
Yield and WUE	Grain yield, yield components, seasonal water consumption	Maturity stage/whole growing season	Plot-wise yield measurement combined with the soil-water balance method

Abbreviations: Pn, net photosynthetic rate; SPAD, soil and plant analyzer development chlorophyll index; Eh, redox potential; TTC, 2,3,5-triphenyltetrazolium chloride; ORP, oxidation–reduction potential; Pt/SCE, platinum/saturated calomel electrode; WUE, water-use efficiency.

**Table 3:** Grain yield, yield components, total water consumption, and water use efficiency (WUE) of rice under nine coupled “irrigation × auxiliary practice” treatments (averaged over 2024–2025).

Treatment	Grain Yield (kg ha^−1^)	Panicles (no. m^−2^)	Paniclelets per Panicle	Filled Grain (%)	1000-Grain wt. (g)	Total Water Use (m^3^ ha^−1^)	WUE (kg m^−3^)
CK	9421.5 d	449	92.3	85.2	26.7	7078.5	1.331
CI1	10,106.2 bc	487	91.8	85.7	26.4	5839.3	1.731
CI2	9348.9 d	455	90.2	84.6	26.9	5870.1	1.593
CI3	9226.6 d	434	92.8	82.4	27.8	5952.8	1.550
CI4	10,273.6 b	472	93.5	86.9	26.8	6057.1	1.696
FMCI	10,464.9 ab	449	97.4	90.3	26.5	5942.6	1.761
FMCG	8976.0 e	447	91.4	84.1	26.1	6812.4	1.318
SACI	10,578.2 a	450	96.8	89.6	27.1	5872.5	1.801
SACG	10,099.5 bc	448	95.2	88.1	26.9	6934.2	1.456

Note: Treatment codes: CK = continuous flooding (control); CI1–CI4 = controlled irrigation (CI) with lower thresholds at 80%, 70%, 60%, and 75% θfc, respectively; FMCI = film mulching + controlled irrigation (CI) (75% θfc); FMCG = film mulching + continuous flooding; SACI = soil amendment + controlled irrigation (CI) (75% θfc); SACG = soil amendment + continuous flooding. θfc = field capacity (volumetric, 38.2% *v*/*v*). Different lowercase letters within the grain yield column indicate significant differences among treatments at *p* < 0.05 (Tukey HSD test); n = 3 per treatment per year, 6 plot-seasons. WUE = grain yield/total water consumption.

**Table 4:** Rhizosphere microenvironment indicators at the milk stage: root TTC reductive activity, rhizosphere redox potential (Eh), and rhizosphere temperature increment relative to CK (mean ± SE, n = 3).

Treatment	TTC Reductive Activity (μg TPF g^−1^ FW h^−1^)	Rhizosphere Eh (mV)	ΔT vs. CK (°C)
CK	284 ± 30 cd	−20 ± 8 c	+0.0 ± 0.2 b
CI1	376 ± 36 b	+45 ± 10 b	−0.3 ± 0.2 b
CI2	312 ± 32 c	+15 ± 9 bc	−0.2 ± 0.2 b
CI3	248 ± 28 d	−35 ± 7 c	−0.1 ± 0.2 b
CI4	384 ± 38 b	+55 ± 11 ab	−0.4 ± 0.2 b
FMCI	392 ± 40 b	+60 ± 12 ab	+1.2 ± 0.3 a
FMCG	168 ± 22 e	−150 ± 15 d	+2.1 ± 0.3 a
SACI	432 ± 44 a	+80 ± 13 a	−0.2 ± 0.2 b
SACG	396 ± 40 b	+30 ± 10 b	+0.0 ± 0.2 b

Note: Eh was measured in situ at 5–8 cm depth using a Pt working electrode (FJA-6 ORP meter) calibrated against a saturated calomel electrode (SCE) with ZoBell standard solution. TTC reductive activity was determined on fine roots (<1 mm diameter, distal 0–10 cm root segments) following the modified 2,3,5-triphenyl tetrazolium chloride method (37 °C, 3 h dark reaction). Rhizosphere temperature was monitored by EM50 dataloggers with Echo-T probes inserted at a depth of 5 cm during the grain-filling stage (5–15 August). TPF = 1,3,5-triphenylformazan; FW = fresh weight. Different lowercase letters within a column indicate significant differences at *p* < 0.05 (Tukey HSD test).

**Table 5:** Cross-method consensus on feature importance for grain yield prediction: Pearson correlation, path coefficient, PLSR-VIP, RF %IncMSE, EN |β|, SHAP |mean|, LIME |mean|, and consensus rank for the seven retained predictors (n = 27).

Feature	Pearson r	Path Coeff.	PLSR VIP	RF %IncMSE	EN |β|	SHAP |mean|	LIME |mean|	Rank
Root volume (milk stage)	0.881	0.39	1.392	42.6%	0.412	0.438	0.426	1
Aboveground DM (milk stage)	0.762	0.30	1.024	31.4%	0.298	0.312	0.298	2
Root surface area (milk stage)	0.637	— ^a^	1.203	28.7%	0.164	0.195	0.189	3
Net Pn (tillering stage)	0.643	0.20	1.013	18.2%	0.187	0.175	0.182	4
SPAD (milk stage)	0.694	0.21	0.868	15.3%	0.143	0.156	0.148	5
Aboveground DM (heading stage)	0.658	— ^a^	0.903	11.8%	0 ^b^	0.108	0.112	6
Root DM (milk stage)	0.731	— ^a^	— ^c^	9.4%	0 ^b^	0.087	0.091	7

Note: Sample size n = 27 (means of two-year plot pairs). Models were fitted under leave-one-block-out cross-validation (LOBO-CV). Path coefficients were estimated by stepwise regression after retaining variables with VIF < 5; superscript ^a^ indicates exclusion due to multicollinearity (VIF ≥ 5). Superscript ^b^ indicates the coefficient was shrunk to zero by elastic-net regularization (α = 0.5, λ = 0.032). Superscript ^c^ indicates exclusion by the PLSR retention criterion. Kendall’s coefficient of concordance among the six independent ranking methods W = 0.871 (χ^2^ = 36.58, df = 7, *p* < 0.01); pairwise Spearman ρ values are shown in Figure 9b. DM = dry matter; Pn = net photosynthetic rate; SPAD = relative chlorophyll content.

## Data Availability

The datasets used and analyzed during the current study available from the corresponding author on reasonable request.

## Supplementary Materials

The following supporting information can be downloaded at: https://www.mdpi.com/article/10.3390/plants15132037/s1, Table S1: Root morphological parameters at the milk stage under nine coupled treatments (mean ± SE, n = 3); Table S2: Predictive performance and convergence diagnostics of four candidate models for rice grain yield under leave-one-block-out cross-validation (LOBO-CV; n = 27); Table S3: Pairwise Spearman rank correlation (ρ) among six independent feature-importance methods for the seven retained predictors of grain yield; Table S4: Treatment-level Jackknife+ conformal prediction (95% prediction intervals, PI) for grain yield based on the random forest (RF) model (n = 27); Table S5: Simulation study: bias–variance behavior, conformal coverage, and outlier-detection power of the diagnostic framework under three sample sizes (n = 27, 50, 100), each with 1000 Monte Carlo replicates.
