# Supplementary material for: Nine Coupled Irrigation–Agronomic Treatments for Water-Saving Rice Production on Albic Soil: An Interpretable Machine-Learning Diagnosis
Source: Plants (Basel). 2026 Jul 1;15(13):2037. doi: 10.3390/plants15132037 (PMC13364089; doi:10.3390/plants15132037)
Supplement: Supplementary file 1 [file plants-15-02037-s001.zip › plants-4382356-supplementary.pdf]

Appendix S

Table S1. Root morphological parameters at the milk stage under nine coupled treatments (mean ± SE, n = 3).

Treatment	Total root length (cm hill^{-1})	Root surface area (cm^2 hill^{-1})	Root volume (cm^3 hill^{-1})	Mean root diameter (mm)
CK	783.8 ± 24.5	635.8 ± 18.3	36.48 ± 3.2 d	2.30 ± 0.10 c
CI1	881.8 ± 28.7	814.7 ± 22.1	67.65 ± 4.9 b	3.00 ± 0.13 b
CI2	851.9 ± 26.3	778.6 ± 20.8	55.27 ± 4.2 bc	2.80 ± 0.12 b
CI3	812.2 ± 25.1	737.8 ± 19.5	44.62 ± 3.7 cd	2.30 ± 0.10 c
CI4	850.9 ± 27.0	830.4 ± 23.5	62.00 ± 4.6 bc	3.00 ± 0.13 b
FMCI	810.4 ± 21.8	823.2 ± 22.9	63.38 ± 4.7 b	3.10 ± 0.14 b
FMCG	802.5 ± 22.4	796.3 ± 21.6	57.31 ± 4.3 bc	3.10 ± 0.14 b
SACI	907.9 ± 31.2	856.1 ± 25.6	80.81 ± 5.8 a	4.10 ± 0.18 a
SACG	957.5 ± 33.5	878.5 ± 26.8	71.81 ± 5.3 ab	3.40 ± 0.15 ab

Note: Root samples were collected by intact root-soil cube excavation (30 × 30 × 30 cm) at the milk stage; roots were washed and scanned (Epson Expression 12000XL, 600 dpi) and analyzed using WinRHIZO Pro 2019. Different lowercase letters within a column indicate significant differences among treatments at $P < 0.05$ (Tukey HSD test). Treatment codes are defined in Table 3.

Table S2. Predictive performance and convergence diagnostics of four candidate models for rice grain yield under leave-one-block-out cross-validation (LOBO-CV; n = 27).

Model	Tuned hyperparameters	LOBO-CV R^2	Train–val. R^2 gap	Convergence at n = 27
Elastic net (EN)	$\alpha = 0.5$; $\lambda = 0.032$	0.796	0.026	Plateaued (converged)
Random forest (RF)	ntree = 1000; mtry = 3 (OOB-tuned)	0.713	0.207	Not yet converged
PLSR	3 latent factors (CV-selected)	0.751	—	Plateaued
Path analysis	Stepwise selection (VIF < 5)	0.728	—	Plateaued

Note: All four models share the same Z-score-standardized feature space (seven predictors retained by 100% in-fold inclusion across three LOBO-CV outer training folds). Convergence was evaluated by learning-curve analysis (n = 9 to 27, step 3, 100 bootstrap resamples per step). The train–validation R^2 gap is reported only for EN and RF, where it provides an interpretable bias–variance signature; PLSR and path analysis use shrinkage/selection mechanisms that do not yield directly comparable gaps. Bootstrap

(1000 resamples) confirmed that the rank of root volume as the top predictor was robust across all four models. EN was selected as the primary inferential model on the basis of its converged learning curve at n = 27, with RF retained as a non-linear complementary reference for SHAP and partial-dependence analyses; because the non-convergence of RF affects its predictive R^2 rather than the stability of its relative feature ranking (verified by bootstrap and simulation), and because the four non-RF consensus methods independently rank root volume first, RF-based attributions were used only to corroborate, never to override, the converged EN analysis.

Table S3. Pairwise Spearman rank correlation (ρ) among six independent feature-importance methods for the seven retained predictors of grain yield.

**Method**	**Path**	**PLSR-VIP**	**RF %IncMSE**	**EN |β|**	**SHAP**	**LIME**	**Top-ranked feature**
Path	1.000	0.800	0.857	0.886	0.871	0.829	Root volume
PLSR-VIP	0.800	1.000	0.829	0.771	0.814	0.800	Root volume
RF %IncMSE	0.857	0.829	1.000	0.900	0.929	0.905	Root volume
EN |β|	0.886	0.771	0.900	1.000	0.886	0.871	Root volume
SHAP	0.871	0.814	0.929	0.886	1.000	0.952	Root volume
LIME	0.829	0.800	0.905	0.871	0.952	1.000	Root volume

Note: All pairwise Spearman ρ values are statistically significant ($P < 0.001$). The minimum ρ = 0.771 (PLSR-VIP vs EN |β|) and the maximum ρ = 0.952 (SHAP vs LIME), indicating high cross-method agreement. The diagonal entries (ρ = 1.000) are self-correlations. The rightmost column confirms that all six methods identify root volume at the milk stage as the top-ranked predictor of grain yield.

Table S4. Treatment-level Jackknife+ conformal prediction (95% prediction intervals, PI) for grain yield based on the random forest (RF) model (n = 27).

**Treatment**	**Observed yield (kg ha⁻¹)**	**LOO predicted (kg ha⁻¹)**	**PI lower (kg ha⁻¹)**	**PI upper (kg ha⁻¹)**	**Half-width (kg ha⁻¹)**	**Coverage**
CK	9421.5	9486.2	9100.0	9872.4	386.2	✓
CI1	10106.2	10024.8	9622.3	10427.3	402.5	✓
CI2	9348.9	9412.6	9026.4	9798.8	386.2	✓
CI3	9226.6	9385.4	8983.2	9787.6	402.2	✓
CI4	10273.6	10198.2	9795.7	10600.7	402.5	✓
FMCI	10464.9	10382.5	9968.3	10796.7	414.2	✓
FMCG	8976.0	9838.7	9251.4	10426.0	587.3	✗ (0/3)

SACI	10578.2	10612.8	10226.6	10999.0	386.2	✓
SACG	10099.5	10056.3	9642.1	10470.5	414.2	✓

Note: RF model fitted with mtry = 3, ntree = 1000 under LOBO-CV. Jackknife+ intervals computed following Barber et al. (2021). "✓" denotes that all three replicate observations of a treatment fall within the 95% PI; "✗" denotes coverage failure. The mean half-width across treatments is 420.2 kg ha^{-1}; SACI yields the narrowest interval (386.2 kg ha^{-1}), whereas FMCG produces the widest (587.3 kg ha^{-1}) and is the only treatment with complete coverage failure (0/3). The empirical coverage rate at the observation level is 88.9% (24/27), slightly below the nominal 95% — a known small-sample behavior of distribution-free conformal methods. The parallel elastic-net (EN) Jackknife+ analysis yields concordant overall results: mean half-width 396.4 kg ha^{-1}, SACI half-width 372.8 kg ha^{-1}, FMCG half-width 568.4 kg ha^{-1} with coverage 0/3, and overall empirical coverage 88.9% (24/27).

Table S5. Simulation study: bias–variance behavior, conformal coverage, and outlier-detection power of the diagnostic framework under three sample sizes (n = 27, 50, 100), each with 1000 Monte Carlo replicates.

Metric	n = 27	n = 50	n = 100
**Feature ranking stability**			
Frequency root volume ranked 1st (EN)	89.4%	96.2%	99.1%
Frequency root volume ranked 1st (RF SHAP)	86.7%	94.8%	98.6%
Top-3 Spearman ϱ (mean ± SD)	0.832 ± 0.118	0.912 ± 0.071	0.964 ± 0.038
**Bias–variance decomposition (EN)**			
LOBO-CV R^2 (mean ± SD)	0.781 ± 0.082	0.803 ± 0.052	0.812 ± 0.031
Train–validation R^2 gap	0.029 ± 0.011	0.018 ± 0.008	0.011 ± 0.005
**Bias–variance decomposition (RF)**			
LOBO-CV R^2 (mean ± SD)	0.689 ± 0.124	0.748 ± 0.087	0.792 ± 0.054
Train–validation R^2 gap	0.213 ± 0.046	0.142 ± 0.032	0.078 ± 0.021
**Jackknife+ empirical coverage (nominal 95%)**			
EN model	88.4% ± 4.2%	92.1% ± 3.1%	94.2% ± 2.4%
RF model	87.6% ± 5.1%	91.4% ± 3.6%	93.8% ± 2.7%
**Permutation-test power (EN model)**			
$\alpha_k = -400$ kg ha^{-1}	0.41	0.62	0.84
$\alpha_k = -800$ kg ha^{-1}	0.78	0.92	0.99
$\alpha_k = -1200$ kg ha^{-1}	0.94	0.99	1.00
**Mondrian group conformal coverage (nominal 95%)**			

m = 3 (per-treatment stratum)	76.4% ± 11.8%	82.6% ± 9.1%	88.5% ± 6.4%
m = 6 (film / amendment groups)	84.7% ± 8.2%	89.3% ± 5.6%	92.4% ± 4.1%
m = 15 (no-auxiliary group)	91.2% ± 5.4%	93.6% ± 3.8%	94.7% ± 2.6%
**EN–RF cross-model concordance (FMCG-like outlier)**			
α_k = −800 kg ha^{-1} (concordant diagnosis)	81.3%	91.6%	97.8%
α_k = −1200 kg ha^{-1} (concordant diagnosis)	94.7%	99.1%	99.9%

Note: Synthetic data were generated with covariance Σ_X estimated from observed data, β set from the elastic-net standardized coefficients, $\alpha_k \in \{-400, -800, -1200\}$ kg ha^{-1}(FMCG-like outlier effect; observed FMCG residual ≈ −860 kg ha^{-1} falls in the α_k = −800 bin), and additive noise calibrated to overall R^2 ≈ 0.78.Because ΣX and β are inherited from the observed data, this table quantifies the reliability boundaries of the diagnostic framework under a realistic data structure and does not constitute out-of-distribution validation of the predictor ranking, which is established independently on the observed data (Sections 3.3 to 3.5). Each (n, α_k) combination was repeated 1000 times with seeds 1–1000. EN = elastic net; RF = random forest; LOBO-CV = leave-one-block-out cross-validation; SHAP = SHapley Additive exPlanations.
